# Insight into the PEC and interfacial charge transfer kinetics at the Mo doped BiVO_4_ photoanodes†

**DOI:** 10.1039/c9ra08743e

**Published:** 2019-12-16

**Authors:** Sriram Kumar, Satyaprakash Ahirwar, Ashis Kumar Satpati

**Affiliations:** Analytical Chemistry Division, Bhabha Atomic Research Centre Trombay Mumbai 400085 India asatpati@barc.gov.in; Homi Bhabha National Institute Anushaktinagar Mumbai 400094 India; Indian Institute of Technology Bombay Mumbai India

## Abstract

BiVO_4_ is a promising photoanode material for the photoelectrochemical (PEC) oxidation of water; however, its poor charge transfer, transport, and slow surface catalytic activity limit the expected theoretical efficiency. Herein, we have investigated the effect of Mo doping on SnO_2_ buffer layer coated BiVO_4_ for PEC water splitting. SnO_2_ and Mo doped BiVO_4_ layers are coated with layer by layer deposition through a precursor solution based spin coating technique followed by annealing. At 5% doping of Mo, the sample (SBM5) shows a maximum current density of 1.65 mA cm^−2^ at 1.64 V *vs.* RHEl in 0.1 M phosphate buffer solution under AM 1.5 G solar simulator, which is about 154% improvement over the sample without Mo (SBM0). The significant improvement in the photocurrent upon Mo doping is due to the improvement of various bulk and interfacial properties in the materials as measured by UV-vis spectroscopy, electrochemical impedance spectroscopy (EIS), Mott–Schottky analysis, and open-circuit photovoltage (OCPV). The charge transfer kinetics at the BiVO_4_/electrolyte interface are investigated to simulate the oxygen evolution process in photoelectrochemical water oxidation in the feedback mode of scanning electrochemical microscopy (SECM) using 2 mM [Fe(CN)_6_]^3−^ as the redox couple. SECM investigation reveals a significant improvement in effective hole transfer rate constant from 2.18 cm s^−1^ to 7.56 cm s^−1^ for the hole transfer reaction from the valence band of BiVO_4_ to [Fe(CN)_6_]^4−^ to oxidize into [Fe(CN)_6_]^3−^ with the Mo doping in BiVO_4_. Results suggest that Mo^6+^ doping facilitates the hole transfer and suppresses the back reaction. The synergistic effect of fast forward and backward conversion of Mo^6+^ to Mo^5+^ expected to facilitate the V^5+^ to V^4+^ which has an important step to improve the photocurrent.

## Abstract 2

BiVO_4_ is a promising photoanode material for the photoelectrochemical (PEC) oxidation of water; however, its poor charge transfer, transport, and slow surface catalytic activity limit the expected theoretical efficiency.

## Graphical abstract

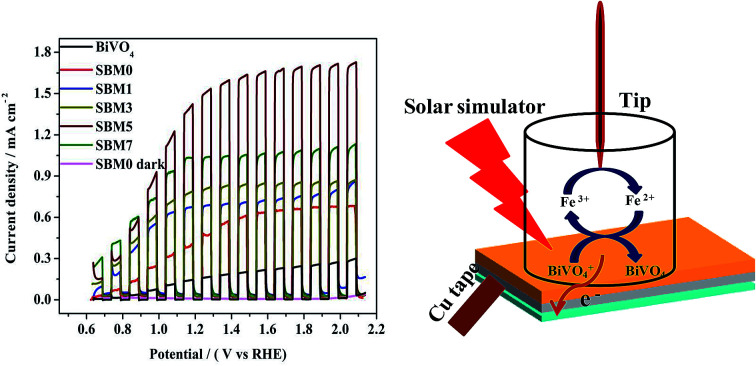

## Introduction

Photoelectrochemical (PEC) splitting of water is one of the most promising methods for simultaneous conversion of hydrogen from water using solar energy as a sustainable and clean energy source and zero carbon footprint; the process has inherently high power and energy densities.^1–5^ PEC water splitting consists of a photoanode and photocathode on which an oxygen evolution reaction (OER) and a hydrogen evolution reaction (HER) respectively are taking place. The overall water splitting reaction, however, is limited by the sluggish OER kinetics; therefore, it is important to develop efficient photoanodes for the improvement in the overall water splitting process. Some of the important materials under continued investigation as photoanode materials for the OER are TiO_2_, α-Fe_2_O_3_, WO_3_ and BiVO_4_.^6–14^ Among these materials, BiVO_4_ is the most researched photoanode due to its suitable band position, bandgap and high theoretical efficiency (∼7.5 mA cm^−2^) and high solar to hydrogen (STH) conversion efficiency (∼9%).^15–18^ However, the slow surface catalytic activity, short hole diffusion length, fast electron–hole recombination are major challenges with the BiVO_4_.^6,19–22^ To overcome these challenges, a number of the strategies have been widely investigated such as; nanostructure control,^23–26^ band engineering,^17,27–34^ heteroatom doping,^35–41^ generation of oxygen vacancy^22,42–44^ and the oxygen evolution catalyst (OEC) incorporation.^18,45–53^ SnO_2_ has been used for the heterojunction formation in the BiVO_4_ system which suppresses the back electron–hole recombination process.^17^ Additionally, the SnO_2_ underneath of BiVO_4_ blocks the surface state of the ITO/FTO. These modifications improve the injection of the photogenerated holes to the electrode–electrolyte interface. During OER at the photoanode surface, photogenerated holes are expected to react with the OH^−^ to form OH˙ radical intermediate, which is converted to O_2_ and very small part of them dimerize to H_2_O_2_ or OH^−^. Some of the OH˙ intermediate diffuse out into the electrolyte. The charge transfer kinetics at the electrode–electrolyte interface is in the range of nanosecond, which is very difficult to investigate by the conventional electrochemical method. Electrochemical impedance spectroscopy (EIS)^54^ and the transient absorption spectroscopy (TAS)^21^ are two important techniques implied to investigate the PEC processes and generate important parameters like photo-induced carrier lifetime and diffusion length.

Scanning electrochemical microscopy (SECM) is a powerful technique to investigate the charge transfer kinetics for *in situ* measurements at the solid–liquid and liquid–liquid interfaces.^55–57^ SECM is decisively applied for the investigation of the mechanism of interfacial charge transfer processes in OER, HER, oxygen reduction reaction (ORR) and hydrogen oxidation reaction (HOR) on the Pt, Pd, Au Hg, and the other electrodes.^56,58,59^ Interfacial charge transfer kinetics at the semiconductor–electrolyte interface has been investigated for the photo-induced charge mediated reactions.^60^ Bard group has demonstrated the detection, quantification, and evolution of decay kinetics of the photogenerated hydroxyl radicals in the PEC on the semiconductor interface.^61,62^ Surface interrogation SECM (SI-SECM) technique has been utilized to quantify photogenerated hydroxyl radicals (ads) and dimerization of the photogenerated radicals at the photoanode.

Considering the shortcoming of BiVO_4_, present investigation is aimed to improve the catalytic efficiency by developing the BiVO_4_ photoanodes with SnO_2_ as interlayer, BiVO_4_ was doped with Mo to further improvement of the catalytic activity. The SnO_2_ coating over the ITO plate was carried out for suppressing the charge recombination process through the generation of heterojunction of SnO_2_ and BiVO_4_. The optical, chemical, and electronic properties of the materials have been investigated to understand the improvement in the PEC efficiency on Mo doping. The improvements in the photocurrent on Mo doping are analyzed based on the relative improvements in the bulk and surface properties as measured by the EIS and Mott–Schottky analysis. The decrease in the charge transfer resistance (*R*_ct_) shows the improvements in the bulk property of the BiVO_4_. The increase in the capacitance upon the Mo doping suggests better activity at the electrode–electrolyte interface due to the enhancement of the active surface sites, which leads to the enhancement in the PEC efficiency.^20^ Strong correlation among the optical property of the material, the open circuit photovoltage (OCPV), and onset potential was discussed in relation with the improvement in the PEC efficiency on Mo doping. The increase in the flat band potential and OCPV suggests the improvements in the charge separation upon the Mo doping which resulted in the enhancement in PEC efficiency.^63–66^ SECM has been applied to investigate the photo-induced interfacial charge transfer kinetics *in situ* at the electrode–electrolyte interface, the interfacial photo-generated hole transfer kinetics was correlated with the efficiency of PEC process across different catalysts investigated.

## Experimental section

### Materials

Bismuth(iii) nitrate (Bi (NO_3_)_3_·5H_2_O, 98%), ammonium vanadate (NH_4_VO_4_, >99%), stannic chloride (SnCl_4_, 98%), and sodium sulfite (Na_2_SO_3_, 98%) were purchased from Sigma Aldrich. Ammonium tetrathiomolybdate ((NH_4_)_2_·MoS_4_, 99.95%) was purchased from Alfa Aesar. Sodium sulphate (Na_2_SO_4_), sodium monohydrogen phosphate (Na_2_HPO_4_), and sodium dihydrogen phosphate (NaH_2_PO_4_) were obtained from Sarabhai M Chemicals. Ethylene glycol and potassium ferricyanide were purchased from SDFCL and used as received.

### Fabrication of photoanode

SnO_2_/BiVO_4_ heterojunction was prepared by the spin coating technique. In this typical synthesis procedure, SnCl_4_ (98%, 0.24 mL, 0.2 M) was dissolved in 10 mL of ethylene glycol and sonicated for 20 min and kept for stirring overnight prior to the spin coating. An aliquot of 100 μL as prepared precursor solution of SnO_2_ was spin-coated on the ITO substrate at 2000 rpm for 1 min, followed by annealing at 250 °C on the hot plate for 5 min. This process was repeated 8 times to get the optimum thickness of SnO_2_.^67^ After spin coating; the modified electrodes were annealed at 450 °C in a tube furnace for 2 h at 5 °C per minute heating rate to form crystalline SnO_2_. After having the SnO_2_ layer over the ITO substrate, the BiVO_4_ film was formed on the ITO/SnO_2_ substrate by metal–organic decomposition method. In this typical synthesis method, Bi(NO_3_)_3_·5H_2_O (0.2 mmol) was dissolved in 5 mL of ethylene glycol–water mixture (8 : 2, volume ratio) subsequently NH_4_VO_3_ (0.2 mmol) was added slowly, the mixture was sonicated for 30 min and kept on stirring for overnight at room temperature. The above solution was spin-coated over ITO (for control experiments) and ITO/SnO_2_ substrate at 2000 rpm for 1 min and then annealed at 350 °C for 5 min on the hot plate. The coating was carried out for repeated 8 times to achieve the appropriate thickness for maximum efficiency,^67^ and after completion of the coating, the samples were heated at 450 °C for 3 h at the heating rate of 5 °C per minute in a tube furnace. Mo doping was achieved by adding ((NH_4_)_2_·MoS_4_, 99.95%) at 1, 3, 5 and 7 atom percentage, resulting in a mixture containing Bi/(V + Mo) = 1 : 1 to replace V position in the crystal lattice of BiVO_4_. The photoanodes thus fabricated using without Mo and at 1, 3, 5 and 7 atom percentage of Mo are designated as SBM0, SBM1, SBM3, SBM5, and SBM7 respectively.

### Photoelectrochemical measurements

Photoelectrochemical measurements were performed using the CH Instrument (920 D model) using a three-electrode cell with an Ag/AgCl (3.0 M KCl) reference electrode, glassy carbon rod as counter and modified ITO coated with the catalyst material as the working electrode. 0.5 M Na_2_SO_4_ solution in 0.1 M potassium phosphate buffer solution (PBS, pH = 7) was used as electrolyte. All photoelectrochemical studies were carried out using Ag/AgCl (3 M KCl) reference electrode and potentials were converted and reported to reference hydrogen electrode (RHE) using the following eqn (1).

1 *E*_(RHE)_ = *E*_Ag/AgCl_ + 0.059pH + *E*^0^_Ag/AgCl_

where, *E*_Ag/AgCl_ is working potential and *E*^0^_Ag/AgCl_ is standard potential (*i.e.* 0.2243 V).

To measure the charge transfer efficiency and the charge transport efficiency, 0.1 M Na_2_SO_3_ as hole scavenger was added in the electrolyte. All samples were front-illuminated because of significantly higher photocurrent than that of back illumination. Solar simulator having 1 sunlight fitted with AM 1.5 G filter was used as a light source. The xenon arc lamp is used as a monochromatic light source; power of the monochromatic light is measured by digital power meter from Newport. The photocurrent was measured by linear sweep voltammetry (LSV) technique with 5 mV s^−1^ scan rate and chopped light voltammetry was recorded. Chronoamperometry was used for the stability test of the photoanode materials. Electrochemical Impedance Spectroscopy (EIS) was used to measure interfacial charge transfer resistance (*R*_ct_) at 1.44 V by applying sinusoidal wave of amplitude 10 mV in the frequency range from 10^5^ to 10^−1^ Hz under 1 sun illumination. Further, the relaxation frequency and time constant of the electrochemical process were measured from EIS data to quantify the efficiency of the photoanode materials. Mott–Schottky experiments were carried out at 100 Hz frequency for the measurements of donor density and flat band potential of photoanode material which inherently affects the PEC activity. Incident photon to current efficiency (IPCE) was measured with the setup similar to that of PEC measurement with monochromatic light from 350 to 650 nm with a 10 nm step. The incident light power was measurement at each wavelength with a calibrated photodiode. SECM study was performed on 920 D bi-potentiostat (CH Instrument) using four electrodes system. SECM Teflon cell was in-house fabricated for holding the substrate at the base with O-ring, reference and counter electrodes. Photoanode materials were used as the substrate, Ag/AgCl (3 M KCl) as a reference and glassy carbon rod as a counter electrode and results are reported in terms of RHE potential. Commercial Pt ultra microelectrode (UME) having RG value of 5 and a diameter 9 μm was used as the probe. Pt microelectrode was polished with a micro polishing cloth with 0.05 μm alumina powder successively and then cleaned in 0.5 M H_2_SO_4_ solution for 20 cycles of cyclic voltammetry scans in the potential window of 1.44 V to 0.29 V *vs.* RHE at the scan rate of 50 mV s^−1^. Ferricyanide solution of 2 mM concentration was used as a redox couple in 0.1 M PBS of pH 7. The potential of the probe was chosen at 0.64 V in the region of steady diffusion current after recording the CV in 2 mM ferricyanide solution with a scan rate of 50 mV s^−1^. Samples were illuminated from the front side using the solar simulator as the light source. Probe Approach Curve (PAC) technique was used to record the approach curve to the substrate in dark and also under the illumination of light to measure the kinetic parameter using four electrodes system at different polarization potentials, from the fitting of the probe approach plots the interfacial charge transfer kinetics were obtained. The mapping of photoanodes was carried out by the SECM technique (constant height mode) under the illumination condition to map the catalytic activity of the catalyst substrate.

### Characterization of the materials

XRD analysis of the prepared samples was performed by using a Rigaku powder diffractometer (9 kW Rotating Anode) with Cu K_α_ radiation (*λ* = 1.5406 A). Raman spectra of photoanodes were recorded by using Lab RAM HR 800 Microlaser Raman system with an Ar^+^ laser of 516 nm. The morphology of the photoanodes was examined by field emission scanning electron microscopy (FE-SEM, JEOL model JSM-7600F). X-ray photoelectron spectroscopy (XPS, MULTILAB, VG Scientific, Al K_α_ radiation as monochromator) was used to investigate the binding energy of the components of Mo doped BiVO_4_ photoanodes.

## Result and discussion

### Structure analysis by XRD and Raman spectroscopy

**Fig. 1:**
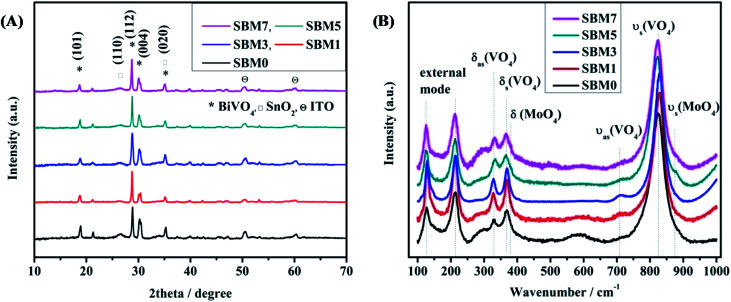
(A) XRD patterns of all BiVO_4_ and Mo doped photoanodes and (B) Raman spectra of all BiVO_4_ based photoanodes.

SnO_2_/Mo-doped BiVO_4_ was as synthesized by sol–gel spin coating method^67^ are characterized by X-ray diffraction (XRD) technique, results are shown in Fig. 1A. XRD peaks at 2*θ* values of 18.59°, 28.61°, 30.01° and 34.96° correspond to (101), (112), (004) and (020) planes respectively of monoclinic phase (JCPDS 75-1867) of BiVO_4_. XRD peaks at 2*θ* values of 26.48° and 50.46° correspond to (110) and (211) plane confirms the presence of inner layer tetragonal SnO_2_ (JCPDS card 77-0450). The presence of these two layers indicates the successful formation of heterojunction. Upon an increase in the atomic percentage doping of Mo, a small shift of peak position towards the lower theta value indicates the expansion of crystal lattice. It has been reported that Mo preferentially substitutes V sites, which is a thermodynamically favorable process, as the impurity formation energy of Mo doping in V sites and Bi sites is 0.53 eV and 2.79 eV, respectively.^68,69^ Moreover, Mo is 6 fold coordinated in MoO_3_, and V and Bi are having 4 folds and 8 folds coordination, respectively. Thus, when Mo atoms substitute V, all Mo–O bonds are expanded; however Mo atoms still keep the 4-folds coordination of V atoms. It has been reported that Mo doping in BiVO_4_ causes deviation of the dipole moment of VO_4_^3−^ tetrahedron from zero to non zero dipole moment in the crystal lattice, thus making the crystal more polar. This enhanced dipole moment due to the distorted polyhedron are reported to promote the charge separation on photo-excitation, which would enhance the overall photocatalytic activity.^69^

Raman spectroscopy is used to investigate the crystallization, local structure and electronic properties of the materials. Fig. 1B shows the characteristic Raman bands of the photoanode materials and tabulated in Table 1. The typical Raman bands of BiVO_4_ are observed at 825.50, 713.80, 367.37 and 329.72 cm^−1^. The strongest band near 825 cm^−1^ is assigned to the *ν*_s_ (VO_4_) (A_g_) and weak shoulder like peak at 713.80 cm^−1^ is assigned to the *ν*_as_ (VO_4_) (B_g_) mode. The Raman band near 367.37 and 329.72 cm^−1^ are assigned to the *δ*_s_ (VO_4_) (B_g_) and *δ*_as_ (VO_4_) (A_g_) respectively. The external modes (rotational and translational) are at lower frequencies than the internal modes of VO_4_^3−^ units because they involve the heavier VO_4_^3−^ unit and having weaker coupling interactions. The modes at 213.23 cm^−1^ and 126.58 cm^−1^ are assigned as rotational and translational modes respectively.^70–75^ The weak bands at 867.50 cm^−1^ and 374.70 cm^−1^ are assigned to the *ν*_s_ (MoO_4_) and *δ* (MoO_4_) respectively. The band corresponds to the *ν*_as_ (MoO_4_) might be covered with 825.50 cm^−1^ peak. All these Raman band assignments for the present materials correspond well with the literature reports of similar materials.^70–75^ It has been observed that there is a blue shift in the most intense peak from 825.50 cm^−1^ to 829.54 cm^−1^ in materials from SBM0 to SBM3 then red shifting is observed in SBM5 to SBM7. The change of vibrational frequency of V–O can be explained in terms of metal–oxygen bond length and strength. F. D. Hardcastle *et al.* have determined the V–O bond length and bond order from Raman stretching frequencies.^75^ Calculations are made based on the correlation of diatomic approximation which assumes that each metal–oxygen bond vibrates independently in the crystal lattice. The empirical formula used for calculation of bond length is as follows^75^

2 *ν* (cm^−1^) = 21 349 exp(−1.9176*R* (Å))

where *ν* is the Raman stretching frequency for V–O in cm^−1^ and *R* is the bond length in Å. It can be seen that the V–O bond varies from 1.696 Å to 1.698 Å upon doping of Mo in BiVO_4_ lattice; thus a marginal increase in the V–O bond length is observed at the 5% Mo content. The asymmetric stretching also has shown similar behavior to that of the symmetric stretching vibrations, where the V–O bond length is decreased initially on the addition of Mo, with further addition of Mo resulted in the increase in V–O bond length. The increase of the V–O bond causes distortion in the lattice, which has resulted in the shifting of XRD peaks towards lower theta values. However, the shifting is not significant in the modification of the crystal structure.

**Table 1:** Analysis of Raman spectroscopy of all BiVO_4_ based photoanodes. All stretching and bending modes of vibrational of BiVO_4_ are listed here. The bond length of V–O is calculated based on the symmetric stretching of V–O mode

Catalysts	*ν*_s_ (V–O) cm^−1^	V–O bond length Å	*ν*_as_ (V–O) cm^−1^	*δ*_s_ (VO_4_) cm^−1^	*δ*_as_ (VO_4_) cm^−1^	Rotational cm^−1^	Translational cm^−1^	*ν*_s_ (MoO_4_) cm^−1^	*δ* (MoO_4_) cm^−1^
SBM0	825.50	1.696	713.80	367.37	329.72	213.23	1286.58	—	—
SBM1	827.54	1.695	710.19	369.37	327.72	213.23	128.18	—	—
SBM3	829.54	1.694	708.19	369.37	327.72	213.23	125.04	867.50	—
SBM5	823.13	1.698	715.80	366.16	330.92	212.30	125.04	867.50	374.70
SBM7	823.94	1.697	716.21	367.37	330.92	212.90	125.64	867.50	374.70

### XPS analysis of the materials

**Fig. 2:**
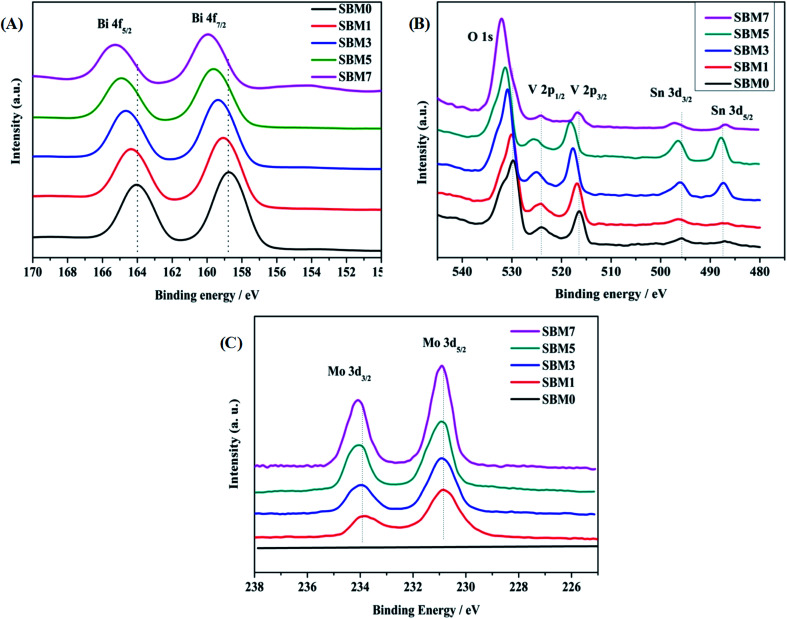
XPS spectra of (A) Bi 4f, (B) O 1s, V 2p and Sn 3d and (C) Mo 3d in BiVO_4_.

XPS was used to investigate the surface electronic properties of photoanodes. As shown in Fig. 2, XPS spectra confirm the presence of elemental constituents of Bi, V, O, Mo and Sn of deposited films on ITO. Fig. 2A shows the spectra of the Bi 4f core level region. The Bi 4f region is characterized by the presence of the 4f_5/2_ and 4f_7/2_ components with a spin–orbit splitting of 5.22 eV. Likewise, V 2p is characterized by the 2p_1/2_ and 2p_3/2_ components with spin–orbit spitting of 7.64 eV as shown in Fig. 2B. There are peaks corresponds to the Sn 3d confirm the presence of the inner layer of SnO_2_ of BiVO_4_. Doping of Mo with varying concentrations has been carried out in BiVO_4_, and corresponding XPS spectra are shown in Fig. 2C. Mo 3d is confirmed by the presence of Mo 3d_3/2_ and Mo 3d_5/2_ spectra with spin–orbit splitting of 3.06 eV, which confirms the doping of Mo in BiVO_4_ lattice, which is in accordance with characterization by XRD and Raman measurements. The surface atomic composition of each sample is calculated using Bi 4f_7/2_, V 2p_3/2_, Mo 3d_5/2_ spectral intensities, weighted by atomic sensitivity. The atomic composition is found to be in good agreement with EDS analysis. Further, in addition to the chemical composition of constituent's elements, chemical states are determined from XPS measurements. Fitting of Bi 4f spectra reveals that each spin–orbit split corresponds to the single oxidation state in all samples. The binding energy positions of Bi 4f_7/2_ and Bi 4f_5/2_ in BiVO_4_ are observed at 158.78 eV and 164.0 eV respectively, and the spin–orbit splitting energy of 5.22 eV suggests that Bi is in +3 oxidation state.^76–79^ The binding energies of V 2p_3/2_ and V 2p_1/2_ are 516.41 eV and 524.05 eV, and spin–orbit split energy is 7.64 eV suggests that V is in +5 oxidation state.^76–79^ All samples exhibit peaks at ∼530 eV for O 1s orbital, which is due to the lattice oxygen. Hydroxyl O 1s peak is also observed near 532 eV suggests the hydroxylated surface of photoanodes, which might enhance the photo-electrocatalysis. Additionally, Mo dopant was characterized by Mo 3d peaks. The binding energies of Mo 3d_3/2_ and Mo 3d_5/2_ are 233.94 eV and 230.88 eV, respectively having spin–orbit split energy 3.06 eV confirming Mo is in +6 oxidation state.^79,80^ The increase of binding energy of all ions (Bi^3+^, V^5+^, O^2−^), in Mo doped BiVO_4_ is the result of doping of higher electronegative dopant (*i.e.* Mo^6+^: 2.16 > V^5+^: 1.63) (Table 2).^81^

**Table 2:** XPS analysis of Bi 4f, O 1s, V 2p and Mo 3d binding energy

Catalysts	Bi 4f_5/2_ eV	Bi 4f_7/2_ eV	O 1s eV	V 2p_1/2_ eV	V 2p_3/2_ eV	Mo 3d_3/2_ eV	Mo 3d_5/2_ eV
SBM0	161.0	158.78	529.80	524.05	516.41	—	—
SBM1	161.34	159.07	529.94	524.21	516.84	233.94	230.87
SBM3	164.70	159.37	530.83	525.10	517.60	233.99	230.92
SBM5	164.94	159.60	531.34	525.36	518.11	234.10	230.93
SBM7	165.27	159.26	531.96	524.21	516.71	234.10	230.90

### SEM and EDS analysis

**Fig. 3:**
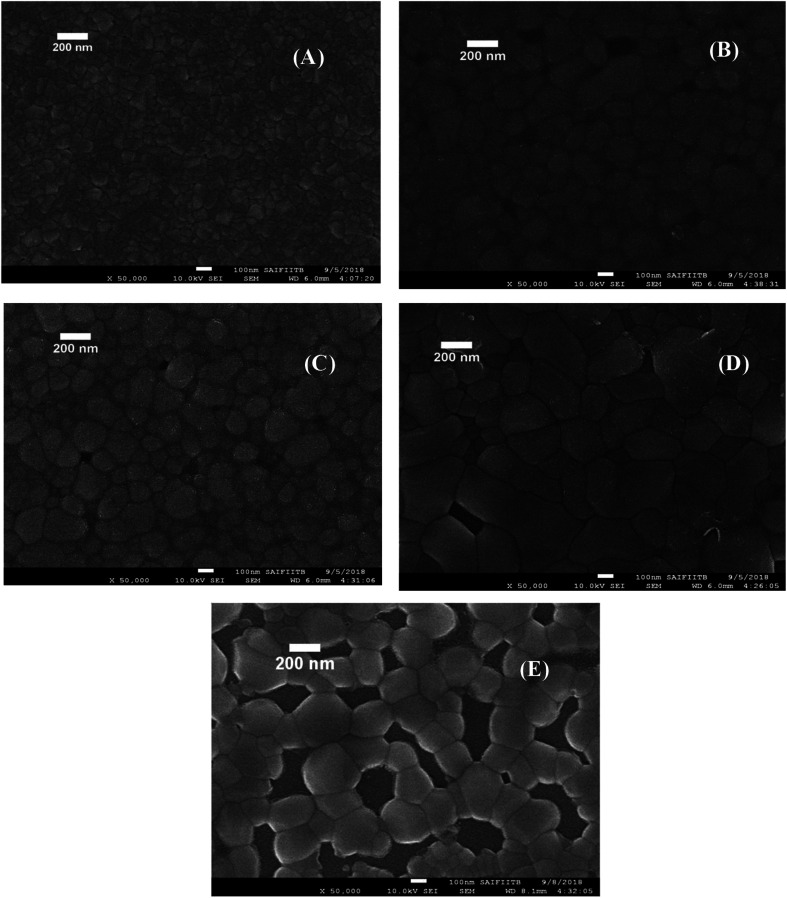
FE-SEM images all porous BiVO_4_ based photoanodes thin films on ITO substrate. (A) SBM0, (B) SBM1, (C) SBM3, (D) SBM5 and (E) SBM7.

Morphology of photoanode materials was characterized by scanning electron microscopy (SEM). BiVO_4_ was uniformly coated on ITO/SnO_2_ as shown in Fig. 3. The materials have shown the granular type of morphology, and the grain size is observed to be increased marginally with Mo doping. Similar observation has been reported in the literature.^82–85^ On 7% Mo doping, the sample has shown chains of grains with vacant spaces in the matrix. Elemental analysis was carried out by energy dispersive spectroscopy (EDS) as shown in Fig. S1 of the ESI.† In SBM0, the atomic ratio of Bi and V is 1 : 1 as shown in Table S1 (ESI†). The atomic percentage of Bi, V, O, and Mo was found as these materials were taken during the synthesis procedure. Sn content corresponding to inner layer SnO_2_ was observed. Further, the thickness of the coating was measured and shown in Fig. S2 (ESI†). The thickness of the combined layers of SnO_2_ and BiVO_4_ was found to be around 440 nm.

### UV-vis spectral measurements

**Fig. 4:**
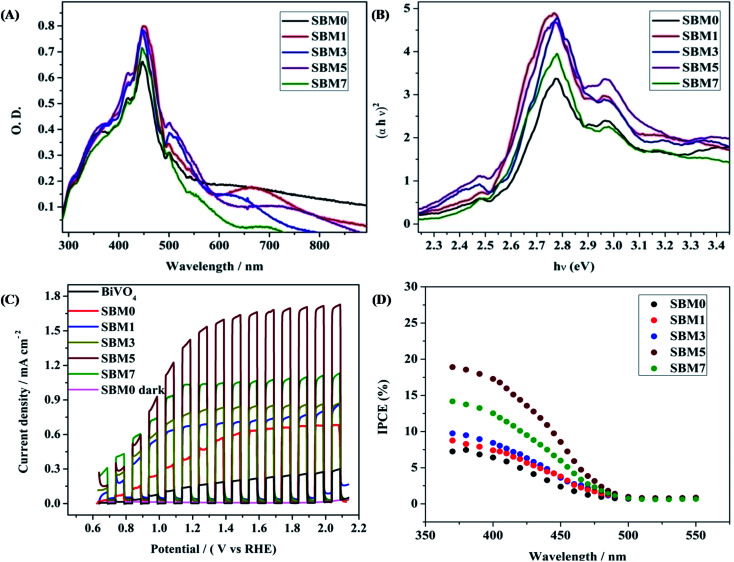
(A) UV-vis diffuse reflectance spectra and (B) Tauc plot for direct band for calculation of bandgap of all BiVO_4_ based photoanodes (C) photoelectrochemical performances of the all BiVO_4_ based photoanodes were measured by chopped light voltammetry in 0.5 M Na_2_SO_4_ in 0.1 M PBS buffer solution at scan rate 10 mV s^−1^ under the 1 sun illuminations. (D) IPCE spectra at 1.0 V *vs.* Ag/AgCl of all photoanodes.

The optical absorption property of a semiconductor reflecting the electronic property of the material is a key factor for determining the photoelectrocatalytic activity. All photoanodes materials are characterized by UV-vis diffuse reflectance spectra of as shown in Fig. 4A. All samples show strong absorption in the visible region, having bandgap absorption edge in the region of 500–550 nm. The most intense absorption peak was observed at 447 nm for BiVO_4_. The absorption bands correspond to the MoO_3_ and SnO_2_ are also observed at 418 nm and 358 nm respectively. A small red shift of absorption edge upon Mo doping is observed. Under the assumption of parabolic band dispersion, the energy dependence of optical transition strength can be explained by Tauc equation^86,87^

3 (*αhν*)^*n*^ = *A*(*hν* − *E*_g_)

Where *α* is optical absorption coefficient, *hν* is photon energy, *E*_g_ is the bandgap and *A* is a probability constant. The numerical values of *n* are 1/2 and 2 for indirect and direct transition, respectively. Thus the nature of the transition can be determined from the linearity of plots of (*αhν*)^1/2^ and (*αhν*)^2^*vs. hν* and bandgap can be determined from the *x*-axis intercept. Fig. 4B shows the Tauc plot for direct band transition. The bandgap of undoped BiVO_4_ is obtained as 2.54 eV. Upon Mo doping, the bandgap is found to decrease marginally from 2.54 to 2.50 eV. On Mo substitution on V site, the band structure remained nearly the same as that of undoped BiVO_4_. The marginal decrease in the bandgap, which means the optical absorption threshold, will not be affected by Mo doping in BiVO_4_.^35,68,69,88^ According to the density of state (DOS), Mo 4d states are mainly distributed in the bottom of VB and CB. Mo atoms have more valence electrons than V and Bi atoms. Thus doping of Mo atoms provides the extra electrons, which cause the rise of the Fermi level. Due to such excess electrons from Mo, impurity energy levels will be formed at the bottom of CB or VB, which would generate more electronic levels; this may lead to better catalytic activity on photoexcitation. The formation of impurity level also generates the broadening of the VB and CB.

### Photoelectrochemical investigation

All PEC experiments were carried out in 0.1 M PBS of pH 7 using 0.5 M Na_2_SO_4_ as supporting electrolyte using three electrodes systems in 1 sunlight source using a solar simulator. Photoresponse of photoanode materials was performed by chopped light voltammetry technique, as shown in Fig. 4C. At 1.64 V (*vs.* RHE) the current densities of 0.65 mA cm^−2^, 0.73 mA cm^−2^, 0.84 mA cm^−2^, 1.65 mA cm^−2^ and 1.06 mA cm^−2^ are obtained for SBM0, SBM1, SBM3, SBM5 and SBM7 respectively. The BiVO_4_ without having the SnO_2_ interlayer has shown the photocurrent of 0.22 mA cm^−2^. The results thus indicate significant improvement in the photocurrent response due to the formation of the SnO_2_ interlayer. The photocurrent is further increased by doping Mo in BiVO_4_. SBM5 has shown the highest photoelectrocatalytic activity. There is ∼154% improvement in the photocurrent from SBM0 upon 5% Mo doping observed in SBM5.

To understand the photoelectrocatalytic activity of BiVO_4_ and Mo doped BiVO_4_, further the incident photon to current efficiency (IPCE) was measured at 1.64 V (*vs.* RHE) in 0.1 M PBS from 370 nm to 550 nm wavelength range, and the results are shown in Fig. 4D. For IPCE measurements, LSV was recorded for the wavelength ranging from 370 nm to 550 nm, and photocurrents were sampled at 1.64 V, and then IPCE was measured by using the following equation^89^4
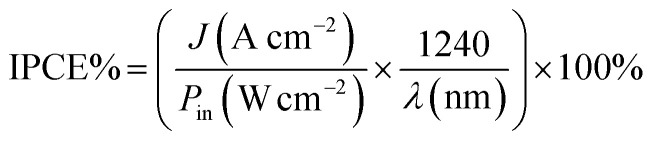
where *J* is the photocurrent density, *P*_in_ is the power of incident photon (monochromatic light), *λ* is wavelength in nm. The onset wavelength of IPCE is 500 nm, which corresponds to the bandgap of 2.48 eV, supports the bandgap calculation from the Tauc plot. On Mo doping IPCE is increased up to the Mo doping of 5%, thereafter on further doping of Mo, no increment is observed. At 5% Mo doping in sample SBM5, IPCE is observed to be at 17% which is about 166% improvements than that of SBM0 at 400 nm.

**Fig. 5:**
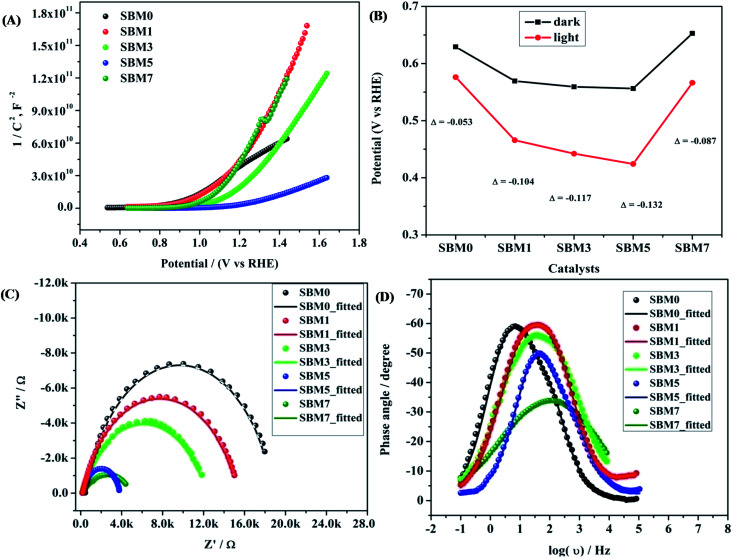
(A) Mott–Schottky analysis for calculation of donor density and flat band potential, (B) OCPV calculation by measuring the difference in potential in dark and illumination, (C) Nyquist plot and (D) Bode plots of the BiVO_4_ based photoanodes.

For further insight into the charge transfer and separation process, Mott–Schottky analysis was carried out; the scans were recorded in the potential window from 0.64 V to 1.64 V (*vs.* RHE) with an increment of 0.025 V at 100 Hz frequency as shown in Fig. 5A.5
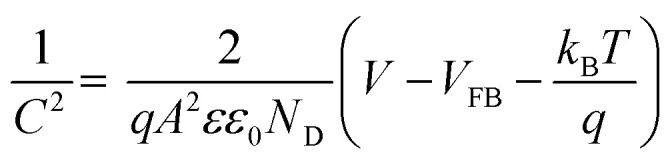
where *C* (F) is space charge capacitance, *q* is elementary charge, *A* is electrode surface area, *ε* is relative permittivity of BiVO_4_ (68),^90^*ε*_0_ vacuum permittivity (8.854 × 10^−12^ Fm^−1^), *N*_D_ (cm^−3^) is donor density, *V* is applied potential, *V*_FB_ is flat band potential and *k*_B_ is Boltzmann constant (1.38 × 10^−23^ J K^−1^) and *T* is absolute temperature. From the slope of a plot of 1/*C*^2^*vs. V*, donor density is measured, which is the inherent property of photoanode materials. Donor density was calculated from the slope of Fig. 5A and tabulated in Table 3. *N*_D_ of SBM0 is found as 5.23 × 10^19^ cm^−3^, the measured value is in accordance with the literature reports.^68,69,88^ Upon doping of Mo, donor density is found to increase, and the values in samples SBM1, SBM3, SBM5, and SBM7 are obtained as 6.24 × 10^19^, 9.66 × 10^19^, 3.85 × 10^20^ and 8.82 × 10^19^ cm^−3^ respectively. This increase of donor density supports the increase of photocurrent with doping of Mo observed in chopped light voltammetry measurements.^68,88^

**Table 3:** PEC activity of the photoanodes were characterized by the following parameters

Catalysts	Band gap (eV)	% increase in photocurrent	Donor density (cm^−3^)	Flat band potential, V	OCPV	% of charge transfer efficiency@1.64 V	% of charge transport efficiency@1.64 V
SBM0	2.54	—	5.23 × 10^19^	0.94	−0.053	46.62	63.73
SBM1	2.52	12.30	6.42 × 10^19^	1.09	−0.014	47.24	78.31
SBM3	2.54	29.23	9.66 × 10^19^	1.16	−0.117	53.90	79.60
SBM5	2.50	153.84	3.85 × 10^20^	1.20	−0.132	50.23	176.27
SBM7	2.53	63.08	8.82 × 10^19^	1.05	−0.087	50.02	95.68

Further, the flat band potential, which is also an important property of photoanode materials and qualitative measurement of the degree of band bending at the electrode–electrolyte interface,^63,64,91^ was calculated from Mott–Schottky plot as shown in Fig. 5A. At higher band bending, the electron–hole recombination will be difficult at the interface, which will result in the improvements of PEC efficiency and stabilizes the photoanodes.^63–65,91^ The flat band potential of SBM0 is found as 0.94 V, on Mo doping, the flat band potential is observed as, 1.09 V, 1.16 V, 1.20 V and 1.05 V for the samples SBM1, SBM3, SBM5, and SBM7 respectively. This observation suggests that the band bending is improved upon Mo doping up to the Mo doping of 5%, which resulted in the suppression of electron–hole recombination on the interface. The suppression of charge recombination facilitates higher charge transfer property at the interface, and hence, overall PEC efficiency is improved. The increase in the flat band potential thus supports the observed enhancement of current in chopped light voltammetry and an increase in the IPCE values on Mo doping up to 5%.

Band bending and charge separation efficiency were further investigated by open-circuit photovoltage (OCPV, *V*_ph_) measurements under AM 1.5 illumination. The extent of band bending is a qualitative measure of in-built potential and charge recombination.^65,66^ The OCPV was calculated from the difference in open circuit potential in dark and illumination as shown in Fig. 5B and tabulated in Table 3 for different samples investigated. OCPV of SBM0, SBM1, SBM3, SBM5 and SBM7 are obtained as −0.053 V, −0.104 V, −0.117 V, −0.132 V and −0.087 V respectively. The shifting of OCPV towards more cathodic side suggests a strong alternation of bands at the electrode–electrolyte interface with Mo doping. This change of OCPV, sourced from the higher band bending under photo-illumination, improves the overall catalytic efficiency by suppressing of charge recombination and improvement of charge separation at the interface. The OCPV is basically the difference between the Fermi levels of the semiconductor when in dark and under illuminated conditions, which is improved on Mo doping. The Fermi level of the semiconductor under illumination, bends to lower its energy, due to which the open circuit voltage differs from that in the dark. The Fermi level bending and the enhancement of OCPV have been schematically shown in Fig. S3 of the ESI.†^65^ On Mo doping the Fermi level undergo enhanced bending, which enhances the OCPV up to the Mo content of 5%. The increase in the OCPV resulted in the improvement of photocurrent on doping of Mo in BiVO_4_.

The interfacial charge transfer efficiency and charge transport efficiency are two limiting parameters on which the overall efficiency of PEC depends. Charge transfer and transport efficiency measurements were performed using a hole scavenger method.^92,93^ LSV was recorded in 0.1 M PBS for water splitting. Hole scavenger Na_2_SO_3_ (0.1 M solution) was used by assuming complete and fast oxidation of sulfite; the LSV plots are shown in Fig. S4 (ESI†). Then, current densities were sampled at 1.64 V and *η*_tranfer_ and *η*_trasport_ were calculated using following equations.6
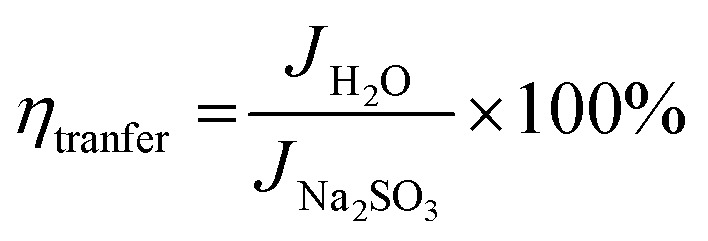
7
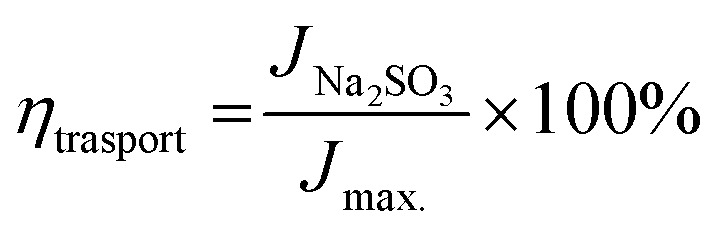
8
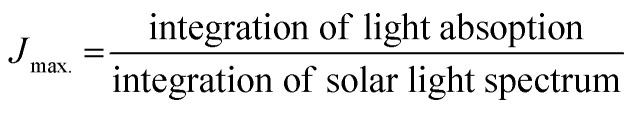
where, *J*_max._ was calculated from the photocurrent obtained from the silicon diode detector. *J*_H_2_O_ is the current density for water oxidation; *J*_Na_2_SO_3__ is the current density for the oxidation of sulfite. Since the water oxidation process is sluggish, sodium sulfite was used as a hole scavenger. It is supposed that oxidation of sulfite is 100%; thus the charge transfer efficiency was calculated with respect to 100% faradaic efficient, *i.e.* how fast charge gets transferred from the electrode–electrolyte interface to water molecule for oxidation. The charge transfer efficiency was calculated and tabulated in Table 3. For SBM0, the charge transfer efficiency is calculated as 46.62%. Doping of Mo in BiVO_4_ resulted in the enhancement of charge transfer efficiency by 15% from the SBM0 sample. The improvement of charge transfer efficiency at the interface is attributed from higher band bending upon Mo doping and increases in donor density as obtained from Mott–Schottky analysis and OCPV measurements. Charge transport efficiency is another factor that greatly affects the photocatalytic activity, it was calculated based on the charge generated inside the photoanode material, and the fraction of it gets transferred at the interface. The charge transport efficiency for SBM0 is obtained as 63.73%. The charge transport efficiency of other samples SBM1, SBM3, SBM5, and SBM7 are 78.31%, 79.66%, 176.27%, and 95.27%, respectively (*cf.*Table 3). These values suggest that band bending upon Mo doping improves charge transport and suppressed the recombination process, as suggested by Mott–Schottky analysis and OCPV values. Thus the improvements of charge transfer and transport efficiencies are the factors responsible for enhancement in the PEC efficiency and support chopped light voltammetry results and IPCE measurements.

### Electrochemical impedance measurements

The conductivity and charge transfer resistance are measured from the analysis of Nyquist plot; it also provides the qualitative insight of the charge transfer processes in bulk as well as at the interface of the photoanode, corresponding results are shown in Fig. 5C. Impedance results are fitted with the equivalent circuit as shown in Fig. S5 (in ESI†) and the fitting parameters are tabulated in Table 4. *R*_ct_ value of SBM0 is found as 16.8 kΩ. When Mo is doped, *R*_ct_ values decreased considerably. The decrease in *R*_ct_ value suggests faster charge transfer at the interface on Mo doping. This variation in *R*_ct_ values supports the PEC activity measurements. The photoanodes were further characterized for their charge relaxation processes from Bode plot analysis as shown in Fig. 5D. Frequencies of phase maxima were sampled for different catalysts, which correspond to the relaxation frequency of photogenerated charge and the results are summarized in Table 4. The relaxation frequency of the SBM0 sample is 6.88 Hz, and the relaxation frequency is increased linearly with the Mo doping from SBM0 to SBM7. Further, the relaxation time constant (*τ*) of the electrochemical process was calculated using *τ* = 1/2π*f* where *f* is relaxation frequency. The decrease in *τ* indicates the faster electrochemical process on Mo doping, which supports the improvements in the PEC efficiency.

**Table 4:** Impedance spectroscopy analysis of photoanodes for measurements of bulk and surface characterization

Catalysts	*R*_ct_ (Ω)	*C*_total_ (F)	Relaxation frequency (Hz)	Relaxation time constant (ms)	*L*_D_ (μm)
SBM0	16820	8.33 × 10^−6^	6.88	23.12	340
SBM1	15060	4.43 × 10^−6^	32.36	4.92	157
SBM3	12010	4.24 × 10^−6^	32.36	4.92	157
SBM5	3610	1.18 × 10^−5^	44.36	3.56	134
SBM7	4832	1.16 × 10^−5^	91.20	1.75	94

Diffusion length is an important parameter in characterizing the interfacial processes, and the overall PEC efficiency depends heavily on the hole diffusion process. The measurement of the diffusion length from impedance measurements, however, includes the assumption that the relaxation time constant is the time taken by the hole to oxidize the water molecule, which contains the diffusion of the hole inside the films and also its diffusion at the electrolyte interface to oxide water molecule. Diffusion length of the photo-generated holes is calculated by using the equation, *L*_D_ = (*D* × *τ*)^2^ where *L*_D_ is diffusion length, *D* is the diffusion coefficient of the photogenerated hole, taken as 0.05 cm^2^ s^−1^,^94^ and *τ* is the relaxation time, the values as obtained are tabulated in Table 4. *L*_D_ of SBM0 is found to be 340 μm. The diffusion length thus obtained from the impedance measurements is decreased with the Mo doping in BiVO_4_. The measured diffusion process includes the diffusion inside the solid catalysts and also at the interface since the observed diffusion length is significantly higher compared to the thickness of the films, the holes are expected to be transported outside the electrochemical interface. The thickness of the films has a negligible contribution to the overall measured diffusion length of the material. The lower diffusion length at the electrochemical interface is associated with the fast charge transfer process at the interface, which is expected to have a higher PEC current.

As seen from Table 4 the capacitance of the SBM0 is significantly low (8.33 × 10^−6^ F) compared to the materials containing Mo. The observed capacitance improvement on the addition of Mo indicates the significant improvement in the surface charge density on the incorporation of Mo in BiVO_4_. These results show that there are surface improvements as well with the improvements in the bulk property of the photoanode upon Mo doping in the BiVO_4_, which enhances the overall PEC efficiency.

### Testing of stability of the photoanodes

**Fig. 6:**
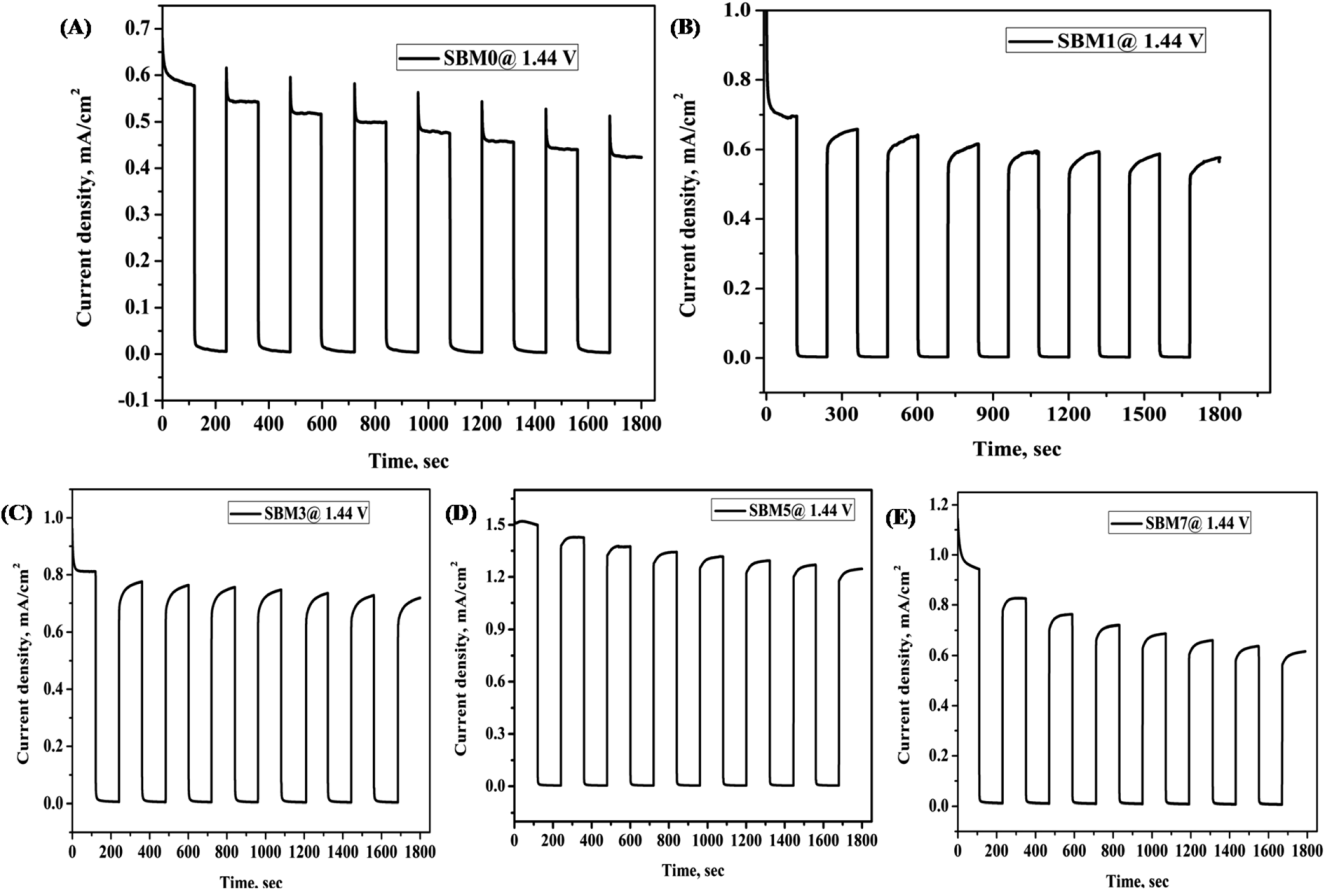
All BiVO_4_ based photoanode was characterized for stability test by chopped light voltammetry at 0.8 V *vs.* Ag/AgCl for 1800 s (A) SBM0, (B) SBM1, (C) SBM3, (D) SBM5 and (E) SBM7.

Stability of photoanode materials is important for prolonged application of the catalyst; it was performed using chronoamperometry technique at 1.44 V *vs.* RHE with chopped light voltammetry method, as shown in Fig. 6, the photocurrent of SBM0 was decreased by 37% after initial excitation. On Mo doping, the recombination step has been reduced drastically, and in place of decay in current, growth in the photocurrent response is observed. The stability test was further performed for continuous illumination of light for 900 s, as shown in Fig. S6 (in (ESI†)) for two catalyst samples. In both, the samples SBM1 and SBM5, the photocurrent is improved initially and then stabilized. The improvement of photocurrent is observed previously and explained on the basis of the charging effect of the photoanode.^95,96^ The photocharging effect has been discussed based on both the surface and bulk modifications in the materials. The redox reaction through the transformation of V(v) to V(iv) is discussed as one of the important reasons behind the enhanced photocurrent due to photocharging. The photocharging effect is observed to be enhanced in the present case on the incorporation of Mo in the catalyst. The reduction potential of V(v) to V(iv) is more positive than the Mo(vi) to Mo(v) reduction; however, the redox kinetics in the later case is significantly faster.^97^ In view of this, during photocharging process, Mo(vi) will get reduced to Mo(v) first due to the kinetic effect; afterward Mo(v) would transfer the electron to V(v) and facilitate the reduction of V(v) to V(iv). As reported previously, this facilitated reduction of V(v) to V(iv) due to the presence of Mo would enhance the catalytic activity on photocharging.^95^ The photocharging effect is also discussed to be due to the interfacial factor through the modification of band structure upon photocharging. The OCPV is measured in all the materials; its value is increased with the Mo doping. The increase in the OCPV can be correlated with the relatively more significant bending of the conduction band than the valence band under photo-illumination. The increase in the OCPV indicates the lesser possibility of recombination and enhancement of the hole transfer property through the interface, which eventually would increase the PEC catalytic process in the material.^65,66^

### Interfacial charge transfer kinetics using SECM

The charger transfer at the semiconductor–electrolyte interface and quantification of the active sites in *in situ* measurements have been performed by the SECM technique.^98–103^ Investigation of the interface has been carried out by the tip feedback mode. When a tip is far from any surface, tip current, *i*_T,∞_ depends on the number of electrons transferred, concentration of electroactive species, diffusion coefficient and radius of ultra-microelectrode.^98^ When the surface is an insulator, the tip current is *i*_T_*i*_T,∞_ which results negative feedback. When surface is conducting, redox active species is generated at the surface, tip current, *i*_T_*i*_T,∞_ as a result, positive feedback is observed.

**Fig. 7:**
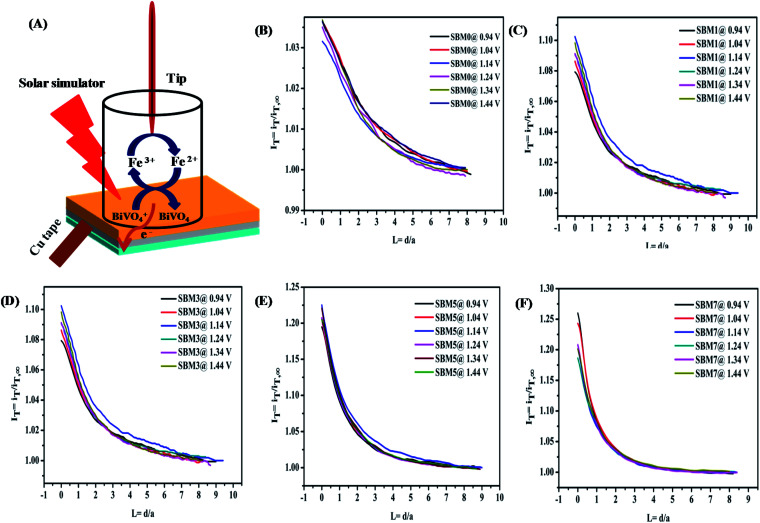
(A) The proposed mechanism of feedback mode of analysis of BiVO_4_/electrolyte interface by SECM analysis. In this technique, a 2 mM ferricyanide solution was used as the redox mediator. In illumination, photo-generated holes oxidizes the [Fe(CN)_6_]^4−^ to [Fe(CN)_6_]^3−^ at the interface and the cathodic potential applied at probe reduces the photo-oxidized [Fe(CN)_6_]^3−^ to [Fe(CN)_6_]^4−^. This redox close-loop gives positive feedback in illumination and negative feedback in dark conditions. SECM imaging of the surface is characterized by the measuring of probe current to measure the hole transfer from the interface to the [Fe(CN)_6_]^4−^. Normalized SECM feedback approach curve of the BiVO_4_ based photoanodes (B) SBM0, (C) SBM1, (D) SBM3, (E) SBM5 and (F) SBM7 in 2 mM [Fe(CN)_6_]^3−^ solution at different applied bias potential at substrate under illumination using Pt ultra microelectrode having *r*_T_ value 4.5 μm as calculated from the cyclic voltammetry measurement shown in (ESI) Fig. S6A.†

In PEC water oxidation reaction, photogenerated holes at the BiVO_4_ interface oxidize water to form oxygen. Since the direct investigation of the water oxidation intermediate or oxygen at the BiVO_4_ surface in the feedback mode is technically difficult, the redox probe [Fe(CN)_6_]^3−^/[Fe(CN)_6_]^4−^ is used rather than the water oxidation intermediate or molecular oxygen.^104^ It has been reported that redox mediator [Fe(CN)_6_]^4−^ and [Fe(CN)_6_]^3−^ can be used as the acceptor of the photogenerated holes and electrons to characterize the redox kinetics of the photo-catalyst for the PEC water-splitting reaction.^105^Fig. 7A shows the schematics of the reaction at the BiVO_4_ interface in the feedback approach. The photogenerated hole at the photoanode/electrolyte interface oxidizes the [Fe(CN)_6_]^4−^ to the [Fe(CN)_6_]^3−^. There is large driving force (Δ*G* ∼ −2.0 eV) for transfer of the photogenerated hole from the valence band of BiVO_4_ to [Fe(CN)_6_]^4−^ for oxidation at the surface catalytic reaction. Therefore, the hole transfer from a valence band of BiVO_4_ to [Fe(CN)_6_]^4−^ is kinetically more favorable by Δ*G* ∼ −0.4 eV than the water oxidation.^106^ The cathodic potential at the probe is tuned to the reduction potential of [Fe(CN)_6_]^3−^ to [Fe(CN)_6_]^4−^ so that there is no side reaction such as oxygen reduction take place. Therefore, feedback current can be assumed mainly from the reduction of [Fe(CN)_6_]^3−^. The hole transfer kinetics at BiVO_4_/interface is measured by the feedback current of the reduction of [Fe(CN)_6_]^3−^ in dark and illumination conditions. In the dark, photoanode behaves as an insulator because of the non-availability of the free electron or hole at the interface and hence negative feedback is observed, however on illumination, it behaves as conducting substrate because of photogenerated holes and electrons. The photogenerated holes at the interface oxidize [Fe(CN)_6_]^4−^ to [Fe(CN)_6_]^3−^, [Fe(CN)_6_]^3−^ species diffuses to the probe and increases the mass transfer process, resulting in the positive feedback response. The normalized tip current (*I*_T_) is calculated by using *I*_T_ = *i*_T_/*i*_T,∞_ where, *i*_T_ is the real time tip current during the approach to the substrate electrode and *i*_T,∞_ is steady current of the tip when the tip is far from the substrate. Prior to the approach of the probe to the substrate, the CV of ultra microelectrode was performed in 2 mM ferricyanide solution as shown in Fig. S7A† shows a good response from the probe. The negative feedback response was fitted using eqn (S1)† for the measurement of RG value. The positive feedback response under illumination was used for the hole kinetics measurements. The positive feedback response of all photoanodes are shown in Fig. 7, the observation of positive feedback indicates the transfer of a hole from the illuminated electrode surface. The normalized apparent heterogeneous charge transfer rate constant (*κ*) and effective heterogeneous charge transfer rate constant *κ*_eff_ (in cm s^−1^) at BiVO_4_/electrolyte interface is obtained from the fitting of experiment approach curve to the theoretical SECM kinetics model using eqn (S1)–(S6).†^107^

All approach curves have been numerically fitted using the above equation and then kinetics parameters are calculated. Some of the fittings of the approach plots are shown in Fig. S8 of the (ESI).† The effective heterogeneous charge transfer rate constant *k*_eff_ is calculated using the relation *k*_eff_ = *κD*_diffusion_/*r*_T_ where *D*_diffusion_ is the diffusion coefficient of the redox probe [Fe(CN)_6_]^3−^ and tabulated in Table 5. The low value of *k*_eff_ for SBM0 shows sluggish hole transfer process at the interface. When Mo was doped in to the BiVO_4_ hole transfer rate constant is found to improve significantly. This improvement suggests that interfacial charge transfer is facilitated upon Mo doping. Further, the effect of applied bias on the hole transfer rate constant is investigated by approaching the probe at different applied potentials. The rate constant for all the materials remained unchanged with the applied potentials indicating its limiting value even at lowest applied potential.

**Table 5:** The *k*_eff_ of hole transfer from the BiVO_4_/electrolyte interface to [Fe(CN)_6_]^3−^

*k*_eff_ (in 10^−3^ cm s^−1^)					
Potential, V	SBM0	SBM1	SBM3	SBM5	SBM7
0.94	2.18	3.58	4.25	6.80	7.50
1.04	2.18	3.90	4.12	7.16	7.48
1.14	2.18	4.25	4.40	7.56	7.16
1.24	2.18	4.0	4.25	7.16	6.48
1.34	2.18	4.0	4.25	7.56	7.16
1.44	2.18	4.25	4.25	7.16	7.16

The localized PEC activity of the BiVO_4_ photoanodes was analyzed by imaging the surface in the constant height mode at the applied potentials of 0.94 V and 1.14 V to the substrate. A cathodic potential of 0.64 V was applied to the probe for the reduction of the [Fe(CN)_6_]^3−^ to [Fe(CN)_6_]^4−^. Therefore, during imaging of the substrate, feedback current at the probe was monitored, and the images are shown in Fig. S9 to S11.† The catalyst substrate is mapped with various current regions, and the current response is increased marginally with more anodic applied potential to the substrate. The overall current response of the tip scanned over the substrate is increased with Mo doping in BiVO_4_ up to 5% doping level. The images are further characterized with bigger current region at doping level up to 5%, originated from the overlapping diffusion layer across the catalyst substrate. At 7% doping, in place of overlapping region, the catalytic currents are characterized by small patches separated from each other. The observation can be correlated from the separated grains at 7% doping compared to the overlapped diffusion layer structure in all other samples. Catalytic current obtained from the overlapping diffusion layer is beneficial in catalyst design, as it provides a similar catalytic activity to that of the continuous films requiring fewer amounts of catalysts. The SECM imaging thus further indicates the betterment in the catalytic activity on Mo doping and also reveals the regional distribution of the catalytic current over the catalyst substrates.

Results thus indicate significant improvement in the catalytic activity of BiVO_4_ through the inclusion of SnO_2_ heterojunction and doping of Mo, which has been explained using some of the important interfacial measurements.

## Conclusion

Present investigation was aimed to improve the photoelectrocatalytic efficiency of BiVO_4_ through the incorporation of SnO_2_ interlayer and doping of Mo. The significant improvement (∼154%) in the photocurrent was observed upon 5% Mo doping in SnO_2_/BiVO_4_. Strong correlation among the optical property of the material, the open circuit photovoltage (OCPV), and onset potential was observed in relation to the improvement in the PEC efficiency on Mo doping. The increase in the flat band potential and OCPV suggests the improvements in the charge separation upon the Mo doping which resulted in the enhancement in PEC efficiency. SECM investigation reveals significant improvement in effective hole transfer rate constant from 2.18 cm s^−1^ to 7.56 cm s^−1^ with the Mo doping in BiVO_4_. The electrochemical impedance investigation supports the improvements in the charge transfer and transport efficiency by improvements in the bulk and surface properties of BiVO_4_. The facilitated reduction of V(v) to V(iv) on Mo doping is also responsible for the improvement in the catalytic activity. The improvement in the catalytic activity has been evaluated from the improvement in the physicochemical at the bulk of the catalysts, its surface, and most importantly due to the improvement in its interfacial charge transfer processes. Mild expansion in the crystal lattice is also observed on replacement of V by Mo, the improvement in the catalytic activity however, related primarily to the electronic nature compared to any morphological changes.

## Abbreviations

SEM: Scanning electron microscope
EDS: Energy dispersive spectroscopy
XPS: X-ray photoelectron spectroscopy
CV: Cyclic voltammetry
LSV: Linear sweep voltammetry
PEC: Photo-electrocatalysis
EIS: Electrochemical impedance spectroscopy
OCPV: Open-circuit photovoltage
UME: Ultra-microelectrode
PAC: Probe approach curve
SECM: Scanning electrochemical microscopy

## Author contributions

The manuscript was written through the contributions of all authors. All authors have given approval to the final version of the manuscript.

## Funding sources

This project is fully funded by our institute Bhabha Atomic Research Centre, Government of India.

## Conflicts of interest

All the authors of this manuscript declare that there is no conflicts of interest exists to disclose.

## Supplementary Material

RA-009-C9RA08743E-s001
