# Supplementary material for: Insight into the PEC and interfacial charge transfer kinetics at the Mo doped BiVO4 photoanodes
Source: RSC Adv. 2019 Dec 16;9(70):41368–82. doi: 10.1039/c9ra08743e (PMC9076381; doi:10.1039/c9ra08743e)
Supplement: RA-009-C9RA08743E-s001 [file RA-009-C9RA08743E-s001.pdf]

Electronic Supplementary Material (ESI) for RSC Advances.
This journal is © The Royal Society of Chemistry 2019

Supporting Information

Insight into the PEC and Interfacial Charge Transfer Kinetics at the Mo Doped $BiVO_4$ Photoanodes

Sriram Kumar[1], Satyaprakash Ahirwar[2] and Ashis Kumar Satpati[1]*

[1]Analytical Chemistry Division, Bhabha Atomic Research Centre, Trombay, Mumbai 400085, India

[1]Homi Bhabha National Institute, Anushaktinagar, Mumbai 400094, India

[2]Indian Institute of Technology, Bombay, Mumbai, India

*Corresponding author: email asatpati@barc.gov.in

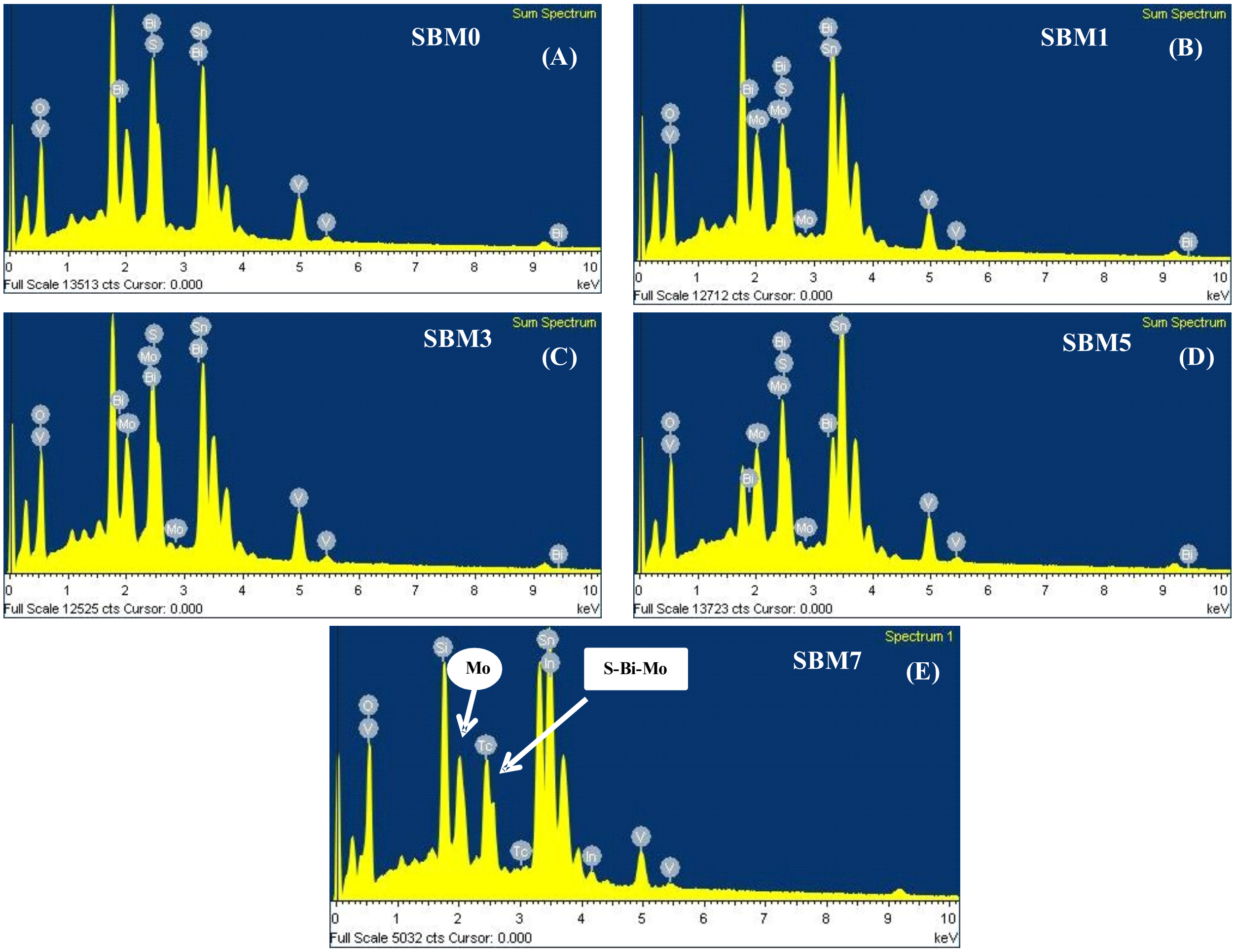

Fig. S1. EDS analysis of different catalysts for calculation of Bi, V, O and Mo in the photoanodes (A) SBM0, (B) SBM1, (C) SBM3, (D) SBM5 and (E) SBM7.

Table S1. Elemental analysis of Bi, V, O, Mo and Sn in $BiVO_4$ photoanodes.

**Elements**	**SBM0**	**SBM1**	**SBM3**	**SBM5**	**SBM7**
	Atomic %	Atomic %	Atomic %	Atomic %	Atomic %
**Bi M**	11.20	10.51	11.14	11.34	10.47
**V K**	11.45	9.54	9.24	8.39	7.34

**O K**	72.06	71.31	69.63	64.49	66.02
**Mo L**	-	0.78	3.18	4.43	6.37
**Sn L**	5.10	6.53	5.57	5.90	9.80

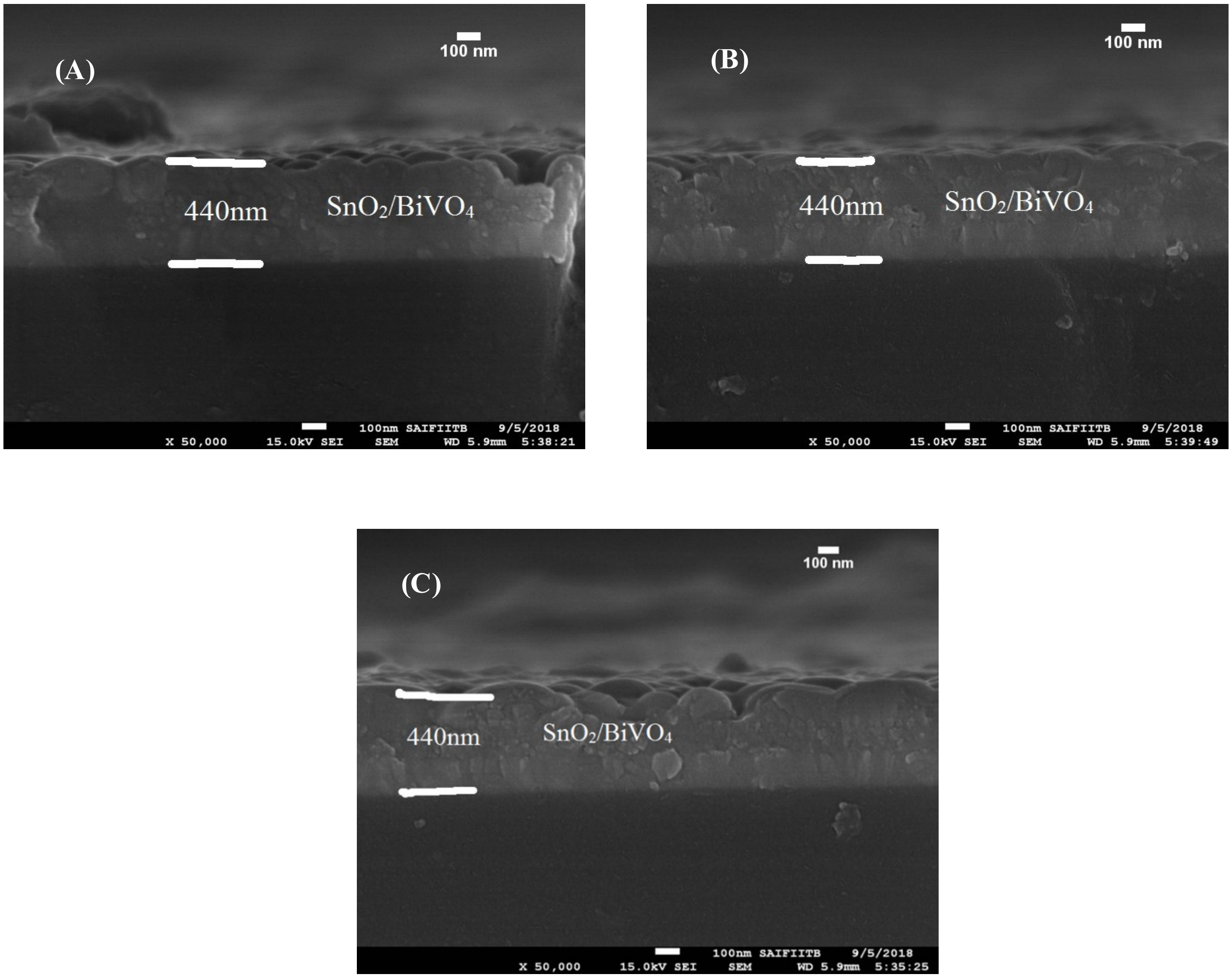

Fig. S2. Cross-sectional view of photoanodes for thickness measurements (A) SBM0, (B) SBM3 and (C) SBM7. Total thickness of film is 440nm

Table S2. The comparative study of the performance of BiVO4 based photoanodes.

Photoanodes	j at 1.23 V vs. RHE ($mAcm^{-2}$) without co-catalyst	j at 1.23 V vs. RHE ($mAcm^{-2}$) With co-catalyst	Buffer/pH	Year	Reference
Mo:$BiVO_4$	2.16	na	Natural sea water. pH = 6	2011	Luo et al.[1]
Mo:$BiVO_4$/CoPi	0.2	1.0	0.5 M Na_2SO_4, pH = 7	2011	Pilli et al.[2]
W:$BiVO_4$/CoPi	1.1	3	0.1 M PBS, pH = 7.3	2013	Abdi et al.[3]
$BiVO_4$/FeOOH/ NiOOH	1.9	4.5	0.5 M PBS, pH = 7	2014	Kim et al.[4]
Mo:$BiVO_4$/FeO OH	1.1	3.0	0.1 M PBS, pH = 7	2014	Park et al.[5]
H_2 treated Mo:$BiVO_4$/CoPi	2.5	4.9	0.1 M PBS, pH = 7	2015	Kim et al.[6]
N_2 treated $BiVO_4$/FeOOH	2.90	5.20	0.5 M PBS, pH = 7.2	2015	Kim et al.[7]
$BiVO_4$/CoO_x/Ni O	1.1	3.5	0.1 M PBS, pH = 7	2015	Zhong et al.[8]
Mo:$BiVO_4$	1.9	na	0.1M PBS, pH = 7.3	2017	Rohloff et al.[9]
ZnO/$BiVO_4$/CoP i	0.28	2.45	0.3 M Na_2SO_4, pH = 7.5	2017	Yang et al.[10]
Mo:$BiVO_4$	2.1	na	0.1 M Na_2SO_3	2018	Huang et al.[11]

$SnO_2/BiVO_4$	2.62	na	0.5 M PBS, pH = 7.	2018	Byun et al.[12]
Fluorinated Mo:$BiVO_4$/CoPi	1.45	5.43	0.1 M PBS, pH = 7.2	2019	Rohloff et al.[13]
BiVO4/Fe-phenolic layer (FTA)	1.5	5.50	0.5 M BBS, pH = 9	2020	Cao et al.[14]
Mo:$BiVO_4$	1.50<br>3.46 (in 0.1 M Na_2SO_3)	na	0.1 M PBS, pH = 7		**This work**

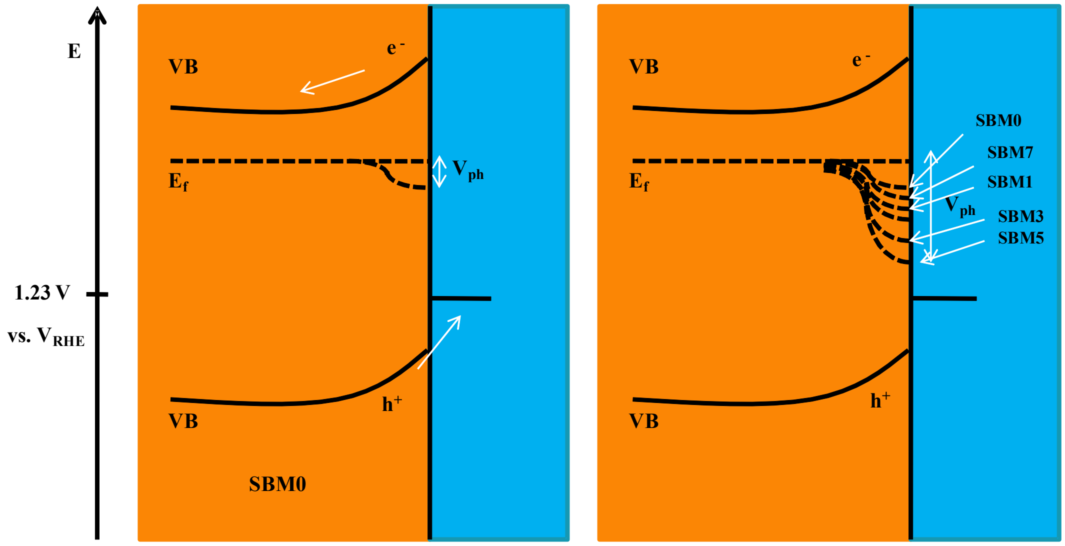

Fig. S3. Band diagram of BiVO4 and Mo doped BiVO4. The band diagram is constracted using UV-vis spectroscopy, OCPV and flat band potential measurements (not o scale).

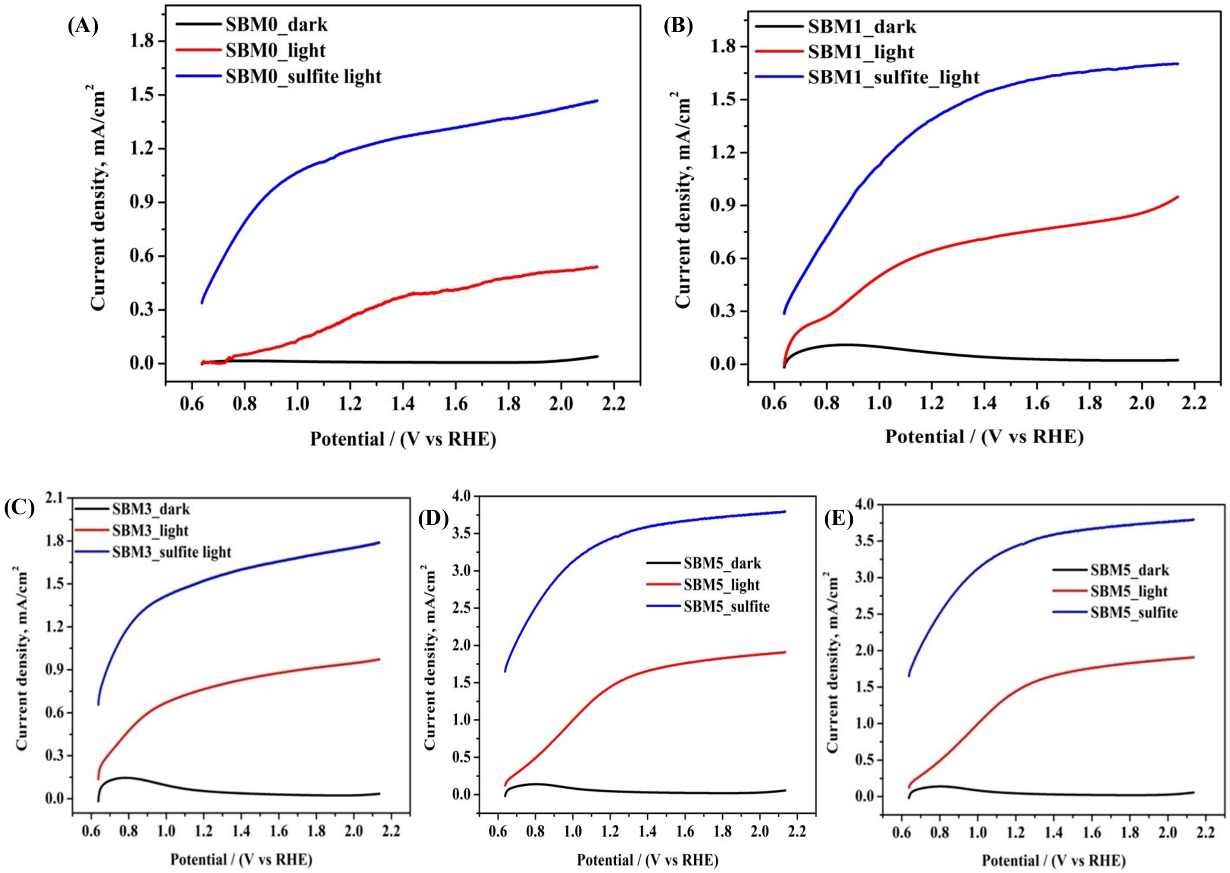

Fig. S4. LSV of photoanodes (A) SBM0, (B) SBM1, (C) SBM3, (D) SBM5 and (E) SBM7 in 0.5M Na_2SO_4 in 0.1M PBS for PEC water oxidation reaction and 0.1M Na_2SO_3 as hole scavenger for charge transfer kinetics measurement.

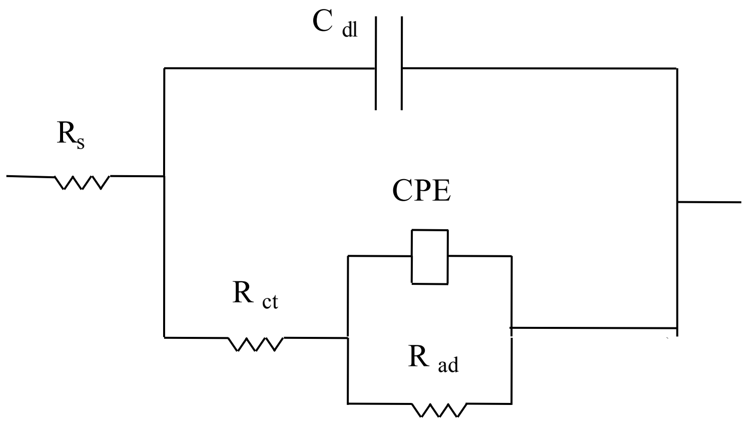

Fig. S5. The equivalent electrical circuit used for fitting the impedance spectroscopy data.

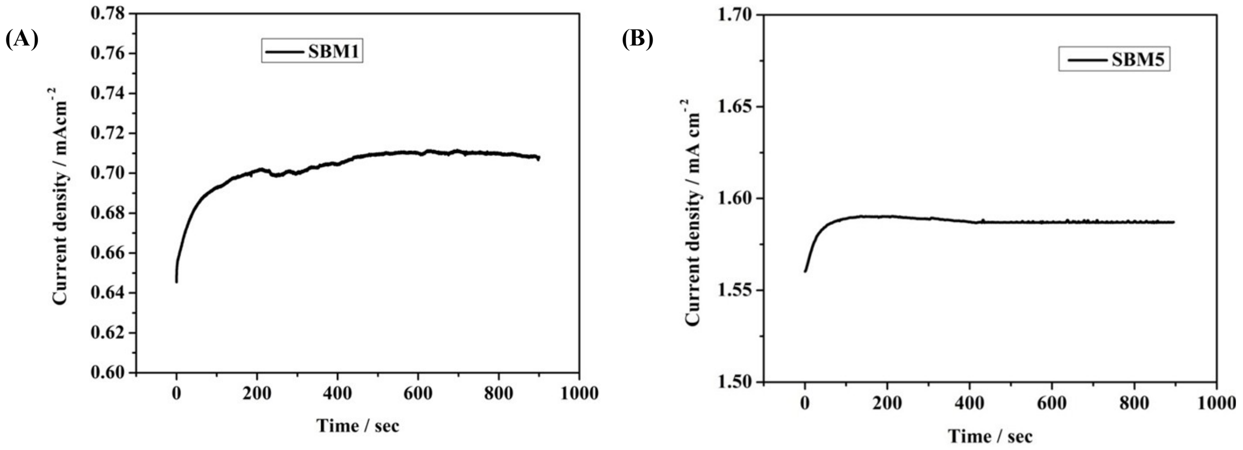

Fig. S6. Stability test of (A) SBM1 and (B) SBM5 in 0.5M Na_2SO_4 in 0.1M PBS at 1.44V.

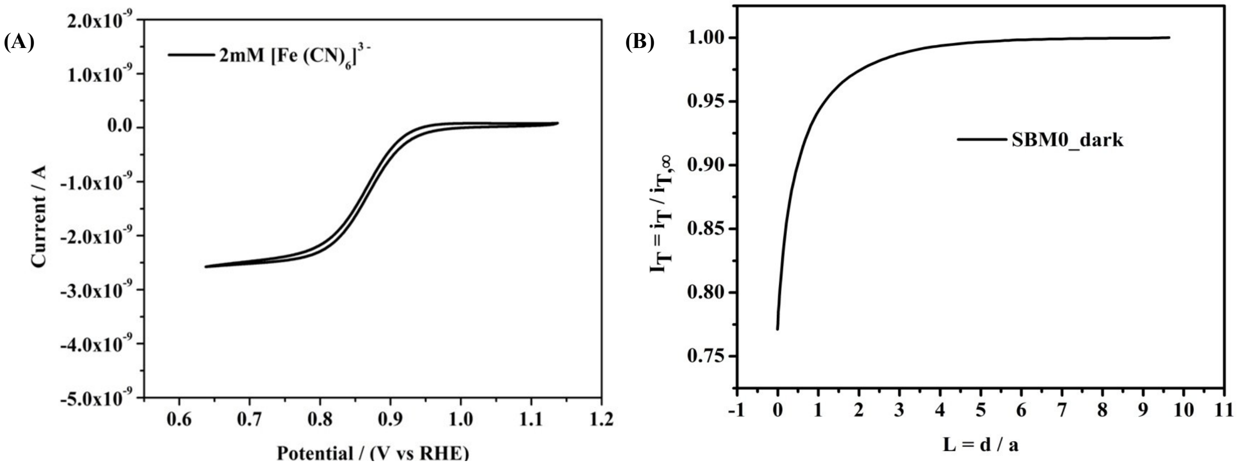

Fig. S7. (A) CV of Pt ultra-microelectrode (UME) in 2mM $[Fe(CN)_6]^{3-}$ at 10mVs $^{-1}$ scan rate and (B) probe approach curve (PAC) of Pt UME in 2mM $[Fe(CN)_6]^{3-}$ solution at SBM0 photoanode in dark.

The normalized approach curves are fitted by using these equations 1-6.[15]

$$I_T^{ins}(L,RG) = \frac{\frac{2.08}{RG^{0.358}}\left(L - \frac{0.145}{RG}\right) + 1.585}{\frac{2.08}{RG^{0.358}}(L + 0.0023RG) + 1.57 + \frac{LnRG}{L} + \frac{2}{\pi RG}Ln\left(1 + \frac{\pi RG}{2L}\right)} \qquad (1)$$

$$I_T^{cond}\left(L + \kappa^{-1}, RG\right) = \alpha(RG) + \frac{\pi}{4\beta(RG)arc\tan\left(L + \kappa^{-1}\right)} + \left(1 - \alpha(RG) - \frac{1}{2\beta(RG)}\right)\frac{2}{\pi}arc\tan\left(L + \kappa^{-1}\right) \quad (2)$$

$$I_T(L,\kappa,RG) = I_T^{cond}\left(L + \frac{1}{\kappa}, RG\right) + \frac{I_T^{ins}(L,RG) - 1}{(1 + 2.47RG^{0.31}L\kappa)(1 + L^{0.006RG + 0.113}\kappa^{-0.023RG + 0.91}} \quad (3)$$

$$I_T = I_s\left(1 - \frac{I_T^{ins}}{I_T^{cond}}\right) + I_T^{ins} \qquad (4)$$

$$\alpha(RG) = Ln2 + Ln2\left(1 - \frac{2}{\pi}arcCos\left(\frac{1}{RG}\right)\right) - Ln2\left(1 - \left(\frac{2}{\pi}arcCos\left(\frac{1}{RG}\right)\right)^2\right) \qquad (5)$$

$$\beta(RG) = 1 + 0.639\left(1 - \frac{2}{\pi}arcCos\left(\frac{1}{RG}\right)\right) - 0.186\left(1 - \left(\frac{2}{\pi}arcCos\left(\frac{1}{RG}\right)\right)^2\right) \qquad (6)$$

Where RG = r_{glass}/r_T is the ratio of the radius of glass sheath (r_{glass}) to the radius of the active area of Pt UME (r_T), I_T^{cond} is diffusion control current for conducting substrate i.e. positive feedback, I_T^{ins} is diffusion control current for insulating substrate i.e. negative feedback.

The details reaction mechanism of PEC regeneration has been developed under the steady-state SECM using $[Fe(CN)_6]^{4-}$ / $[Fe(CN)_6]^{3-}$ redox probe on the photoanode [16, 17].

$$FTO/BiVO_4 + hv \rightarrow FTO/BiVO_4^* \qquad (7)$$

$$FTO/BiVO_4^* \rightarrow FTO/BiVO_4^+ + e^-(FTO) \qquad (8)$$

$$FTO/BiVO_4^+ + [Fe(CN)_6]^{4-} \rightarrow FTO/BiVO_4 + [Fe(CN)_6]^{3-} \quad (9)$$

$$[Fe(CN)_6]^{3-} + e^- \rightarrow [Fe(CN)_6]^{4-} \quad \text{at the tip} \quad (10)$$

On solving the above equations using steady-state approximation, the following result has been obtained as

$$\frac{1}{I_s} = \frac{1}{I_{T,\ cond}} + \frac{4D_{diffusion}[Fe^{3+}]^*}{\pi r_T l[BiVO_4^0]\varphi_{hv}J_{hv}} + \frac{4D_{diffusion}}{\pi r_T l[BiVO_4^0]k_{ox}^{'}} \quad (11)$$

For the first-order reaction at the $BiVO_4$ electrode surface, the following expression is in correlation with the feedback approach curve [18, 19].

$$\frac{1}{I_s} = \frac{1}{I_{T,\ cond}} + \frac{1\,1}{\pi\,\kappa} \quad (12)$$

$$k_{eff} = \kappa \frac{D_{diffusion}}{r_T} \quad (13)$$

Experimental normalized probe approach curves are fitted using equations 1-6 at different κ and interfacial effective rate constant (k_{eff}) is calculated using equation 13. The mapping of $BiVO_4$ photoanodes was performed by measuring tip current in feedback mode at constant height to quantify the localized surface catalytic activity in PEC.

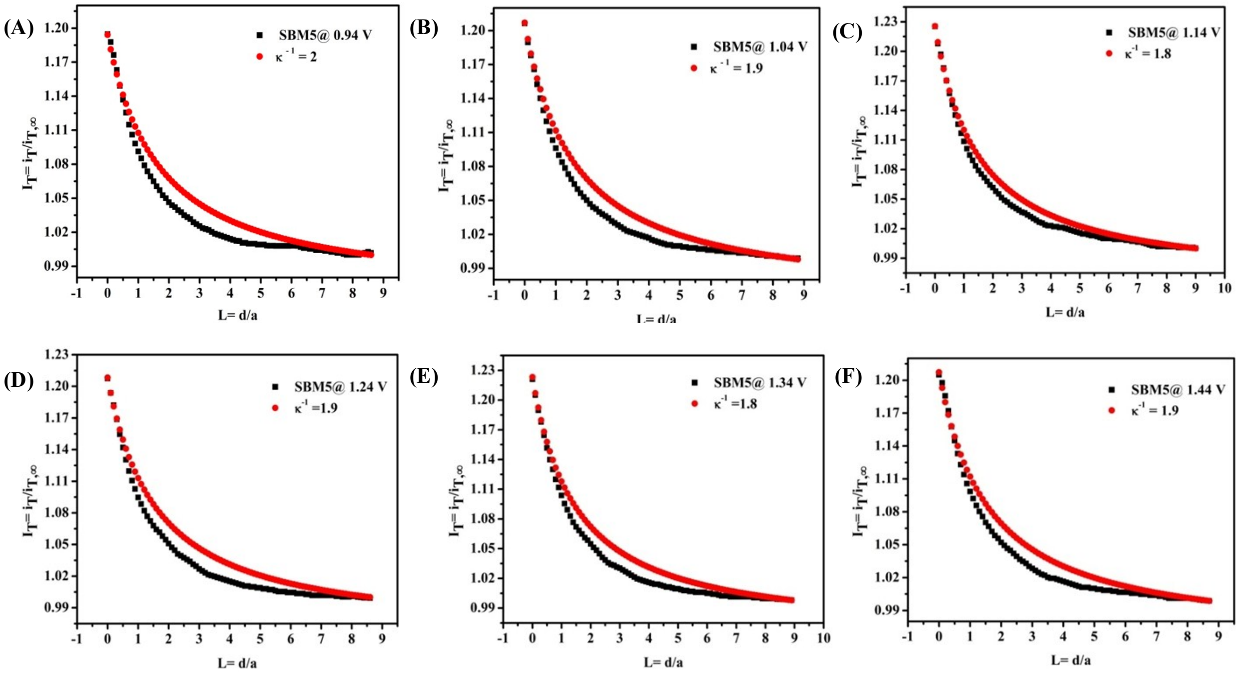

Fig. S8. Normalized SECM feedback approach curve of the SBM5 at (A) 0.94V, (B)1.04V, (C) 1.14V, (D) 1.24V, (E) 1.34V and (F) 1.44V in 2mM $[Fe(CN)_6]^{3-}$ solution at different applied potential at substrate under illumination using Pt ultra-microelectrode having r_T value 4.8μm as calculated from the cyclic voltammetry measurement shown in Supporting Information Fig. S5. The feedback approach curve are fitted with a theoretical model of SECM using equations 8-13 given in the manuscript.

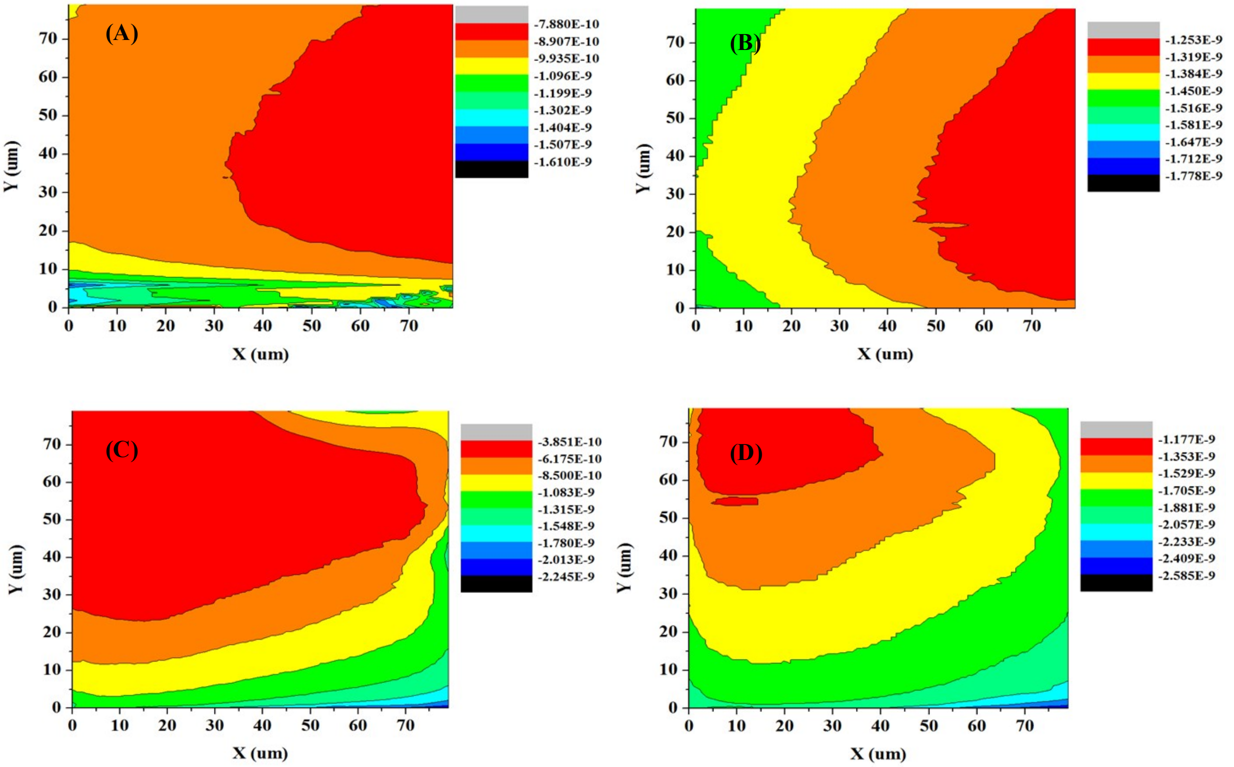

Fig. S9. Scanning electrochemical microscopy (SECM) imaging of photoanodes SBM0 at (A) 0.94V and (B) 1.14V and SBM1 at (C) 0.94V and (D) 1.14V substrate potential in 2mM $[Fe(CN)_6]^{3-}$ at constant height mode using Pt UME as probe and photoanodes as substrate.

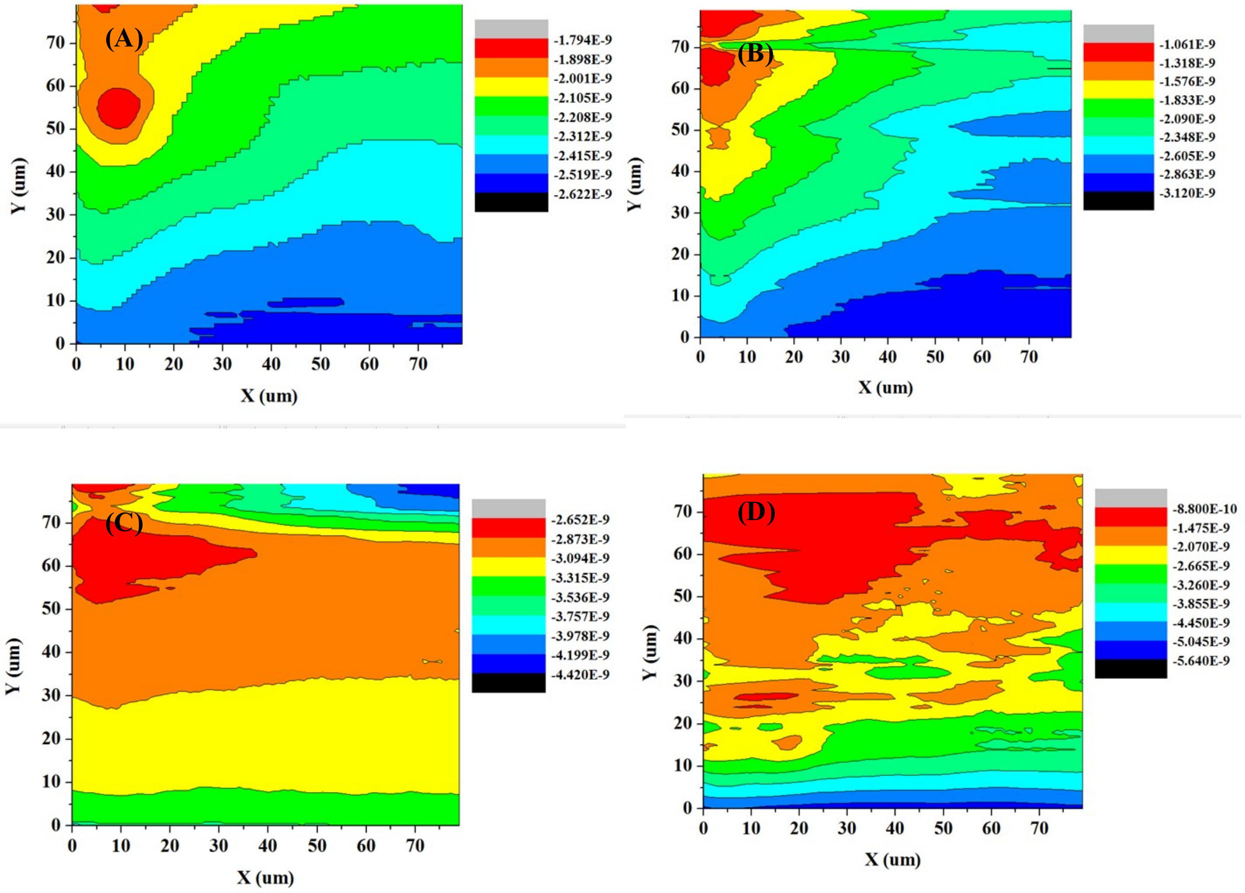

Fig. S10. Scanning electrochemical microscopy (SECM) imaging of photoanodes SBM3 at (A) 0.94V and (B) 1.14V and SBM5 at (C) 0.94V and (D) 1.14V substrate potential in 2mM $[Fe(CN)_6]^{3-}$ at constant height mode using Pt UME as probe and photoanodes as substrate.

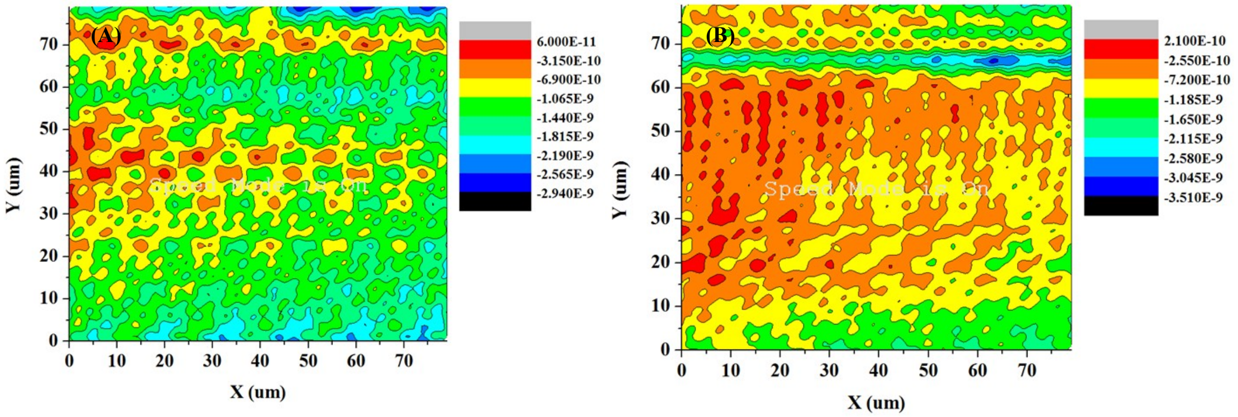

Fig. S11. Scanning electrochemical microscopy (SECM) imaging of photoanodes SBM7 at (A) 0.94V and (B) 1.14V substrate potential in 2mM $[Fe(CN)_6]^{3-}$ at constant height mode using Pt UME as probe and photoanodes as substrate.

Reference

1. W. Luo, Z. Yang, Z. Li, J. Zhang, J. Liu, Z. Zhao, Z. Wang, S. Yan, T. Yu and Z. Zou, *Energy & Environmental Science*, 2011, **4**, 4046-4051.

2. S. Pilli, T. Furtak, L. Brown, T. Deutsch, J. Turner and A. Herring, *J. Am. Chem. Soc*, 2011, **133**, 18370-18377.

3. F. F. Abdi, L. Han, A. H. Smets, M. Zeman, B. Dam and R. Van De Krol, *Nature communications*, 2013, **4**, 2195.

4. T. W. Kim and K.-S. Choi, *Science*, 2014, **343**, 990-994.

5. Y. Park, D. Kang and K.-S. Choi, *Physical Chemistry Chemical Physics*, 2014, **16**, 1238-1246.

6. J. H. Kim, Y. Jo, J. H. Kim, J. W. Jang, H. J. Kang, Y. H. Lee, D. S. Kim, Y. Jun and J. S. Lee, *ACS nano*, 2015, **9**, 11820-11829.

7. T. W. Kim, Y. Ping, G. A. Galli and K.-S. Choi, *Nature communications*, 2015, **6**, 8769.

8. M. Zhong, T. Hisatomi, Y. Kuang, J. Zhao, M. Liu, A. Iwase, Q. Jia, H. Nishiyama, T. Minegishi and M. Nakabayashi, *Journal of the American Chemical Society*, 2015, **137**, 5053-5060.

9. M. Rohloff, B. Anke, S. Zhang, U. Gernert, C. Scheu, M. Lerch and A. Fischer, *Sustainable Energy & Fuels*, 2017, **1**, 1830-1846.

10. J.-S. Yang and J.-J. Wu, *Nano Energy*, 2017, **32**, 232-240.

11. M. Huang, J. Bian, W. Xiong, C. Huang and R. Zhang, *Journal of Materials Chemistry A*, 2018, **6**, 3602-3609.

12. S. Byun, G. Jung, S.-Y. Moon, B. Kim, J. Y. Park, S. Jeon, S.-W. Nam and B. Shin, *Nano energy*, 2018, **43**, 244-252.

13. M. Rohloff, B. r. Anke, O. Kasian, S. Zhang, M. Lerch, C. Scheu and A. Fischer, *ACS applied materials & interfaces*, 2019.

14. X. Cao, C. Xu, X. Liang, J. Ma, M. Yue and Y. Ding, *Applied Catalysis B: Environmental*, 2020, **260**, 118136.

15. R. Cornut and C. Lefrou, *Journal of Electroanalytical Chemistry*, 2008, **621**, 178-184.

16. B. Zhang, X. Zhang, X. Xiao and Y. Shen, *ACS applied materials & interfaces*, 2016, **8**, 1606-1614.

17. U. Mengesha Tefashe, K. Nonomura, N. Vlachopoulos, A. Hagfeldt and G. Wittstock, *The Journal of Physical Chemistry C*, 2012, **116**, 4316-4323.

18. C. Wei, A. J. Bard and M. V. Mirkin, *The Journal of Physical Chemistry*, 1995, **99**, 16033-16042.

19. C. Lefrou and R. Cornut, *ChemPhysChem*, 2010, **11**, 547-556.
